# Adaptive ionic moieties for electrolyte structural design

**DOI:** 10.1126/sciadv.aeh0276

**Published:** 2026-09-25

**Authors:** Oliver S. Hammond, Guillaume Bousrez, Stuart J. Brown, Sichao Li, Daniel C. Morris, Serena Cozzolino, Manishkumar Shimpi, Liliana de Campo, Andrew E. Whitten, Alexei Vorobiev, Andrew Nelson, Jason B. Harper, Anja-Verena Mudring, Oleg N. Antzutkin, Sergei Glavatskih, Mark W. Rutland

**Affiliations:** ^1^ European Spallation Source, Data Management & Scientific Computing, Asmussens Allé 305, Kongens Lyngby 2800, Hovedstaden, Denmark.; ^2^ Division of Surface and Corrosion Science, KTH Royal Institute of Technology, Brinellvägen 8, Stockholm 10044, Stockholms län, Sweden.; ^3^ Department of Biological and Chemical Engineering, Aarhus University, Åbogade 40, Aarhus N 8200, Midtjylland, Denmark.; ^4^ Department of Materials and Environmental Chemistry, Stockholm University, Svante Arrhenius väg, Stockholm 11418, Stockholms Län, Sweden.; ^5^ School of Chemistry, University of New South Wales, Kensington, Sydney 2033, NSW, Australia.; ^6^ Wallenberg Initiative Materials Science for Sustainability, Stockholm, Sweden.; ^7^ Department of Physics and Astronomy, Materials Physics, Uppsala University, Uppsala 75120, Upplands, Sweden.; ^8^ Chemistry of Interfaces, Luleå University of Technology, Universitetsområdet, Luleå 97187, Norrbotten, Sweden.; ^9^ Australian Nuclear Science and Technology Organisation, ANSTO, New Illawarra Rd, Lucas Heights 2234, NSW, Australia.; ^10^ Institut Laue-Langevin (ILL), Avenue des Martyrs, Grenoble 38000, Isère, France.; ^11^ Department of Physics, Umeå University, Linnaeus väg 20, Umeå 907 36, Västerbottens län, Sweden.; ^12^ School of Engineering, University of KwaZulu-Natal, 91 Ridge Rd, Pietermaritzburg 3201, KwaZulu-Natal, South Africa.; ^13^ Unit of Systems & Component Design, KTH Royal Institute of Technology, Brinellvägen 83, Stockholm 11428, Stockholms län, Sweden.; ^14^ Department of Electromechanical, Systems and Metal Engineering, Ghent University, Technologiepark 131, Ghent 9052, East Flanders, Belgium.; ^15^ Laboratoire de Tribologie et Dynamique des Systèmes, École Centrale de Lyon, Écully, Lyon 69134, Auvergne-Rhône-Alpes, France.; ^16^ Bioeconomy and Health Department, Materials and Surface Design, RISE Research Institutes of Sweden, Drottning Kristinas väg 61, Stockholm 10044, Stockholms län, Sweden.

## Abstract

Ionic liquids (ILs) are now an established and ubiquitous material class. In energy applications and electroresponsive lubrication, their interfacial self-assembly is crucial for their properties. This in turn is determined by chemical structure and a local hierarchy of Coulombic, van der Waals, and solvophobic interactions. We hypothesize that delocalization of the ionic charge permits the Coulombic interactions to adapt to other self-assembly imperatives, promoting both bulk and interfacial self-assembly structures and enhancing double layer responses. Novel chelated bis(catecholato)borate anion architectures are introduced, with [P_66614_] cations. Small/wide-angle x-ray scattering of the ILs indicates pronounced cation assemblies. In the electrolyte solvent propylene carbonate, small-angle neutron scattering shows marked anion-dependent self-assembly modulation. Neutron reflectivity reveals analogous response in the electrochemical double layer; the catechols form thicker and more electroresponsive interfacial layers. Density functional theory calculations demonstrate electronic resonance, modulating the anion polarizability. This direct self-assembly control broadly affects electrochemistry, lubrication, and formulation design.

## INTRODUCTION

Developments in fields such as ionic liquids (ILs; defined as low-melting salts where *T*_m_ < 100°C) offer unrealized opportunities for chemical problem-solving, via the “design” of task-specific solvents (*1*) that can benefit from decreased toxicity, flammability, and volatility compared to conventional molecular analogues. Yet, the breadth of chemical space ILs occupy, and complexities involved in their manufacture, means that they are by no means a green panacea (*2*). As the IL research field has matured to ∼100,000 published articles, major issues plaguing popular systems, such as expense and HF liberation, are gradually being resolved (*3*). This has enabled the development of thousands of patents, and a number of successful industrial processes based on IL chemistry, with some mainstream examples including synthesis, electrochemistry, catalysis, extraction, and recycling (*4*).

Enormous environmental and economic impacts can therefore be realized through IL design, with lubrication providing one such case study (*5*, *6*). While the science of lubrication involves many approaches to preventing surface contact, reducing wear and friction, a key component is the deployment of so-called boundary lubricants. These are self-assembled interfacial films that resist expulsion in a tribological contact and prevent direct surface contact, as a “last line of defense.” They also provide a well-defined sliding plane, and have a self-healing ability (*7*). ILs have garnered much attention here because they cannot only form this type of boundary film but also ameliorate the issue of tribocharging. This is the buildup of interfacial charge in lower dielectric media under sliding, the discharging of which is deleterious for the material properties, and which is prevented by self-regulation in the IL boundary films due to the conservation of electroneutrality, thus removing this wear route (*8*, *9*).

Modern expectations of machine efficiency, longevity, and performance necessitate effective contact lubrication, which has been successfully demonstrated with a “double-edged sword” approach of unifying boron chemistry and surface active ionic liquids (SAILs) (*8*, *10*–*12*). SAILs display surface activity, where amphiphilic moieties induce self-assembly phenomena at interfaces (*13*), with the interplay between strong electrostatics and the requirement to maintain charge neutrality causing distinct structures such as layers and chequerboards (*14*). Once adsorbed at the interface, SAILs reduce interfacial friction, but are subject to transient extremes of pressure and temperature (*15*); the enhanced thermal stability of phosphonium cations is thus favored in the context of inevitable degradation. Being mindful of degradation pathways (*10*) allows the realization of this philosophy: upon decomposition, chelated orthoborate anions liberate B species directly at the interface, where they rapidly react to form passivating tribofilms of materials such as B_2_O_3_ and metal borides, protecting against further wear (*16*). The correlation between bulk structure and the surface activity of orthoborate-based ILs using the anions bis(oxalato)borate [BOB]^−^, bis(mandelato)borate [BMB]^−^, and bis(salicylato)borate [BScB]^−^ has been characterized by various techniques (*17*–*20*), providing a structural basis for the observed reduction in friction and wear when orthoborate ILs are deployed as lubricant materials (*21*). Building in complexity, these ionic lubricant materials have now been realized as “tribotronic” agents (*22*). By understanding the interfacial structuring and charge distribution in the cation and anion, the application of potentials controllably alters the structure at the interface, which alters the electrochemical double layer (EDL), and thus, the viscoelastic properties of the contact; (*15*, *18*, *23*, *24*) active control over friction and wear is thus attainable with a holistic approach (*9*). Orthoborate ILs are one such example where tribotronic control via interfacial phase change is directly achievable (*25*). The knowledge gap is therefore closing between well-understood and highly configurable aqueous self-assembly systems (*26*–*28*) and these more esoteric nonaqueous ionic analogues (*29*, *30*).

The same properties that make a benevolent and tribotronically active IL can also be leveraged for the development of halogen free electrolytes for energy storage technologies. Specifically, supercapacitors can greatly benefit from an electrodependent self-assembly structure, and it has been shown that SAILs can be used to improve capacitance and tune electrochemical properties (*13*, *31*, *32*). Supercapacitors are used for grid stability with renewable energy sources, emergency power systems, electric vehicles, and internet-of-things devices (*33*, *34*). Therefore, with modern concerns over the health effects of poly-fluorinated alkyl substances (“forever chemicals”) (*35*) and ever-growing energy demands, supercapacitors will be one of the key technologies enabling sustainable technological development. We highlight tribology and supercapacitor applications as the main beneficiary fields of this research; however, other key fields that rely on solvophobic self-assembly and liquid nanostructure include detergents, drug delivery, liquid-liquid extractions, and catalysis (*36*, *37*). Thus, the ability to selectively tune and enhance this phenomenon has the potential to generate broad impact.

**Fig. 1.**
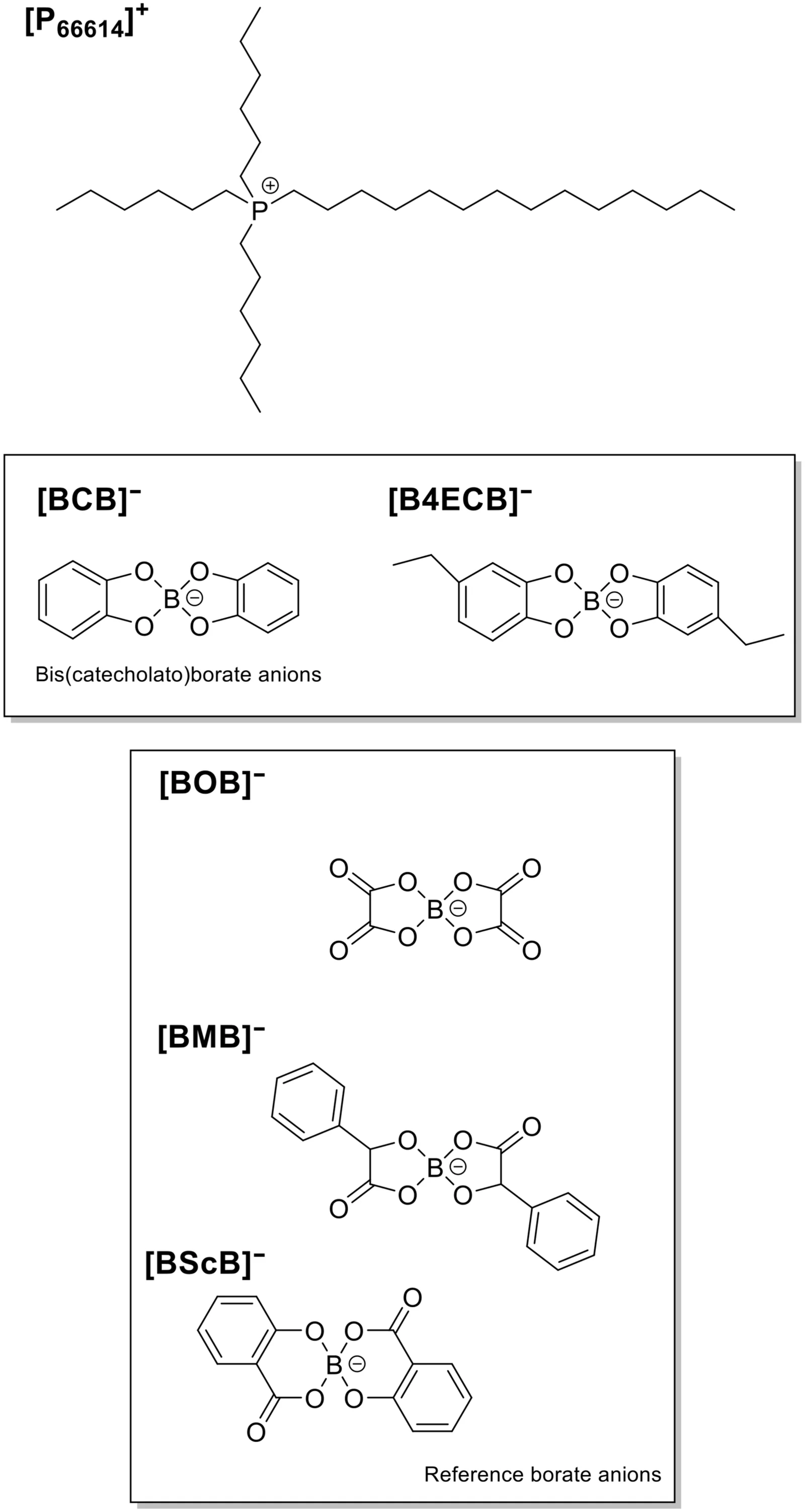
Ionic liquid structures. Structures of the cation [P_66614_]; the reference orthoborate anions [BOB], [BMB], and [BScB]; and the bis(catecholato)borate anions [BCB] and [B4ECB], which are described herein.

Here, we therefore designed new ILs with boron-containing anion chemistries based on bis(catecholato)borate [BCB] structures (see Fig. 1), mixed with the “universal liquefier” [P_66614_] cation (*38*). [BCB] salts were first reported in 1998 as unexpectedly stable reaction side products, but are to our knowledge hitherto unexplored as IL components (*39*). In this manuscript, we present our discovery that this seemingly subtle anion modification causes marked changes in the bulk structure and physical properties of the ILs. Using density functional theory (DFT) calculations, we show that the softened catechol chemistry provides an ever-correct “jigsaw piece” of electrostatic potential where it is needed, thus controlling the dimensions of self-assembled structures, particularly in nonaqueous solvents pertinent to electrochemical systems. Detailed small-angle scattering and neutron reflectometry (NR) experiments demonstrate this ability, and contrast it with the behavior of known orthoborate anions. We show that [BCB] ILs can act as self-contained aggregation modifiers with function analogous to salicylate in aqueous cationic surfactant systems, negating the need for further salts or additives. These findings constitute a breakthrough in terms of the ashless properties critically required for high-temperature lubrication and general formulation, but moreover in the development of “designer electrolytes” with intrinsically tailored surface coverage, mobility, and capacitance (*40*).

## RESULTS AND DISCUSSION

### Anion effect on bulk ILs

Details of the preparation of the [BCB]-based ILs are provided in the Supplementary Materials alongside their characterization. Throughout this work, the two anions bis(catecholato)borate [BCB] and bis(4-ethylcatecholato)borate [B4ECB] will be compared with three well-known “reference” orthoborates; [BOB], [BMB], and [BScB].

**Fig. 2.**
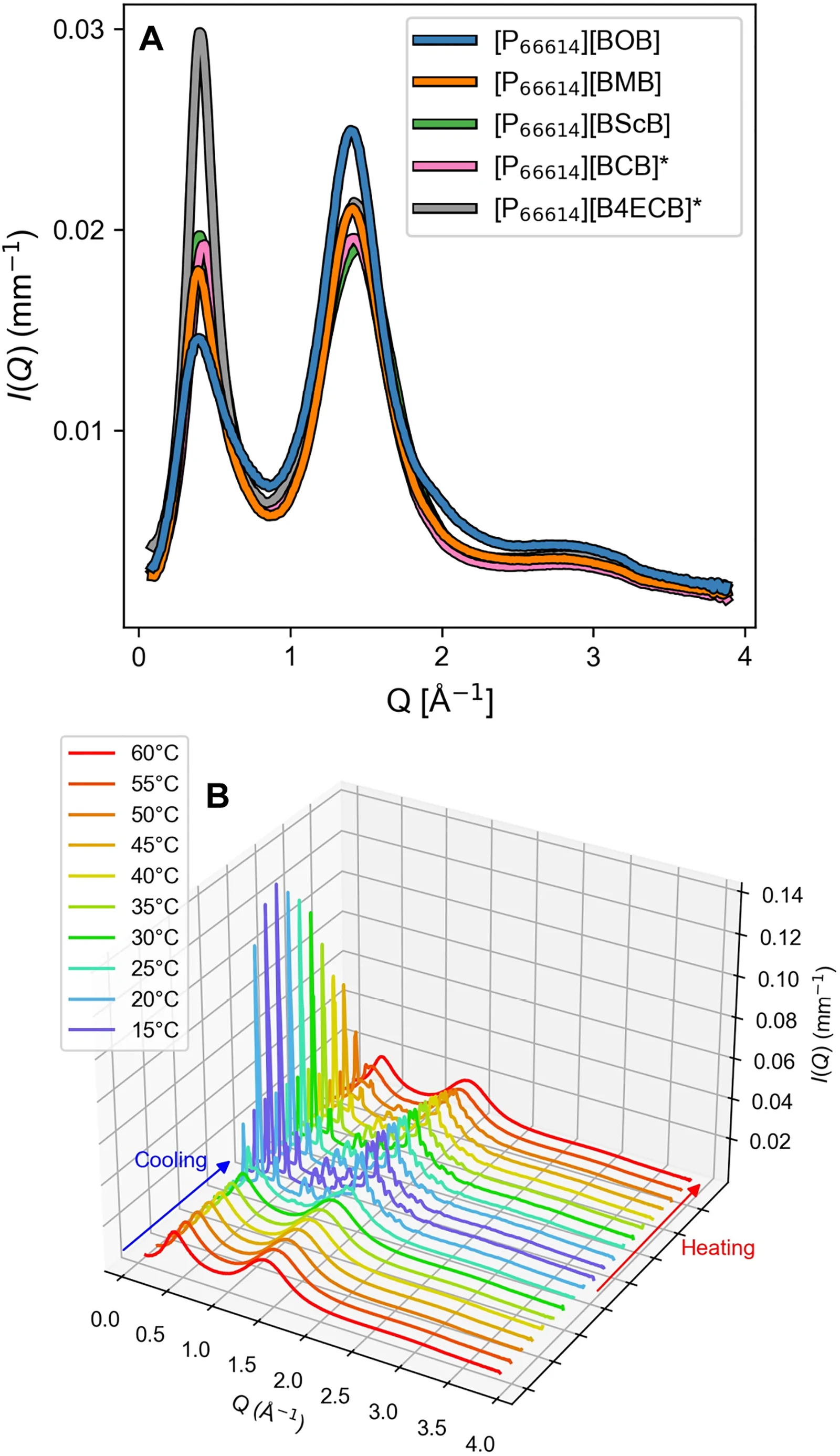
SWAXS data of neat ILs. (**A**) Experimental SWAXS data, where measurements taken at elevated temperatures (50°C) to ensure fluidity are denoted by an asterisk (*) and were otherwise taken at 25°C. (**B**) SWAXS temperature scan measurements of [P_66614_][BCB].

The catecholates display slightly higher melting points (*T*_m_) than the reference species, with [BCB] melting slightly above room temperature. More detailed thermal property investigations revealed some behavioral complexities for the two new catecholate ILs. In terms of stability, [P_66614_][BCB] decomposes at 462°C in a one-step event, whereas [P_66614_][B4ECB] decomposes in two steps, the first at 227°C (13% mass loss) and the second at 415°C (full degradation). As shown in Fig. 2B, temperature-dependent small/wide-angle x-ray scattering (SWAXS) measurements confirmed thermotropic effects for [P_66614_][BCB]. Under controlled supercooling conditions for this IL, it is possible to observe the onset of a highly ordered phase, which could be indexed to a lamellar structure with a *d* spacing of 35.155 ± 0.122 Å; this is followed by more complete crystallization as cooling continues. Complete details of thermal properties are provided in the Supplementary Materials, namely, discussion of thermal history effects and comprehensive DSC (figs. S6, S13, and S14), TGA (figs. S7 and S15), and the temperature-controlled SWAXS analysis (fig. S16). Overall, the thermal behavior is less eventful than that observed for other state-of-the-art orthoborate anion chemistries, such as bis(butane-1,4-dioxy)borate ([B(bdo)_2_]−) and tetrakis(pyrazolyl)borate ([B(pyr)_4_]−), which were shown to be organic ionic plastic crystal formers when paired with a smaller symmetric [P_4444_] cation (*41*).

Beyond basic phase behavior, SWAXS clarifies the nanoscale effects of anion modification (*36*). ILs have stronger local order than molecular liquids, which is assigned to an *L*_3_-type (disordered bilayer spongelike) nanostructure, as was recently demonstrated for various pure “reference” orthoborate ILs using small-angle neutron scattering (SANS) (*19*). While there is very little difference in peak position and, hence, size, the SWAXS data in Fig. 2A show a clear increase in intensity for the low-*Q* feature (termed the polar-nonpolar peak, PNPP; *d*_1_, or other similar names) (*42*–*44*) going from [BOB] to the catecholates, while the short-range close contact adjacency peak (CP; *d*_3_) intensity decreases. These phosphonium orthoborate ILs also display a very high-*Q* broad “*d*_4_” feature that is not often resolved (∼2.8 Å^−1^), while the mid-*Q d*_2_ peak is likely convoluted with the PNPP, as shown for alkyl-decorated phosphonium and ammonium SAILs (*45*). These data therefore suggest that, while the common cation is the main structure-former responsible for hydrophobic domains of a similar size, the anions may play a role in densifying this packing: The integral of the PNPP as a fraction of all integrated peaks (provided in table S1) provides a trend series for this behavior: [BOB] < [BMB] < [BScB] < [BCB] < [B4ECB], where the [B4ECB] has the most profound low-*Q* peak.

### Structure of ILs in nonaqueous media

**Fig. 3.**
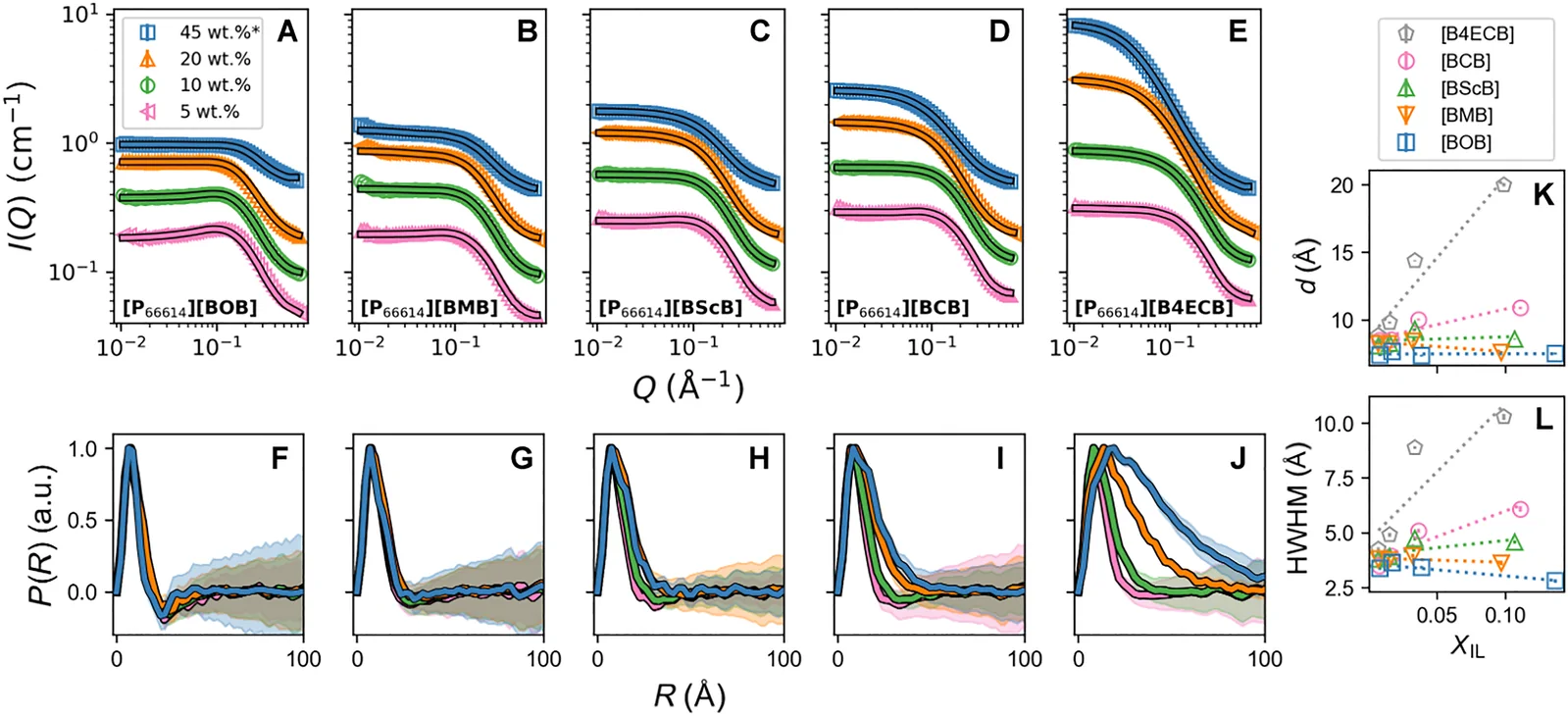
SANS data and analysis of dispersed ILs. (**A** to **E**) Small-angle neutron scattering (SANS) data (markers) for the respective ILs dispersed in 1,2-propylene-*d*_6_-carbonate as a function of concentration, and fits (colored lines) to the data from IFT analysis; (**F** to **J**) corresponding real-space pair distribution functions, *P*(*R*), calculated from the IFT fits (note the common *x*/*y*-axis scaling); (**K** and **L**) peak position and breadth from Lorentzian fits to the *P*(*R*)s, as a function of IL mole fraction (*X*_IL_). *For the most concentrated samples, the median of 45 wt % is stated, with comprehensive sample composition details provided in the Supplementary Materials.

The polar aprotic model electrolyte, 1,2-propylene-*d*_6_ carbonate, was chosen for SANS studies of solution behavior. The same size and self-assembly trend series from bulk SWAXS was observed for these IL dispersions (see Fig. 3, A to E). The SANS *I*(*Q*) (especially at higher concentrations) flattens at progressively lower *Q* as one goes down the anion series, and have increasing *I*_0_, indicating that the catecholate ILs display a higher aggregation number. Indirect Fourier transformation (IFT) provides a series of real-space pair distribution functions, *P*(*R*) (*46*, *47*), which quantify this extension behavior (see Fig. 3, F to J). The *P*(*R*) peak centers (Fig. 3K) shift to longer length scales and simultaneously broaden (Fig. 3L) with asymmetric tailing toward longer lengths, which is characteristic of the formation of longer 1D (i.e., more cylindrical) morphologies (*48*); this effect begins in earnest with [BScB] and is strongest for [B4ECB]. This structuring is resilient to application-pertinent contaminants: 1 wt % D_2_O was added to our samples (see fig. S18), which made almost no discernible difference to the measured forward scattering intensity or form factor.

More detailed fitting was performed using a cylindrical scattering form factor model (*49*), convoluted with the Hayter-Penfold Rescaled Mean Spherical Approximation (RMSA) *S*(*Q*) to describe the interparticle interactions by invoking the DLVO theory; this *S*(*Q*) interpretation is functional, but caveated in applicability here due to the high concentrations of nonspherical aggregates (*50*, *51*). The shape-based model fits are shown in fig. S17 alongside the corresponding parameters. We have favored an approach with fewer freely varied parameters to avoid overfitting, but highlight that more detail may be extracted from the data, such as some core-shell character with a solvating corona, and more nuanced directional ion-ion interactions beyond the capability of the RMSA structure factor (*52*). Nevertheless, all analysis routines point to the same clear conclusion: Catecholate anions cause an outsize increase in aggregate size and encourage 1D growth.

### The electrochemical double layer

#### General interfacial nanostructure

**Fig. 4.**
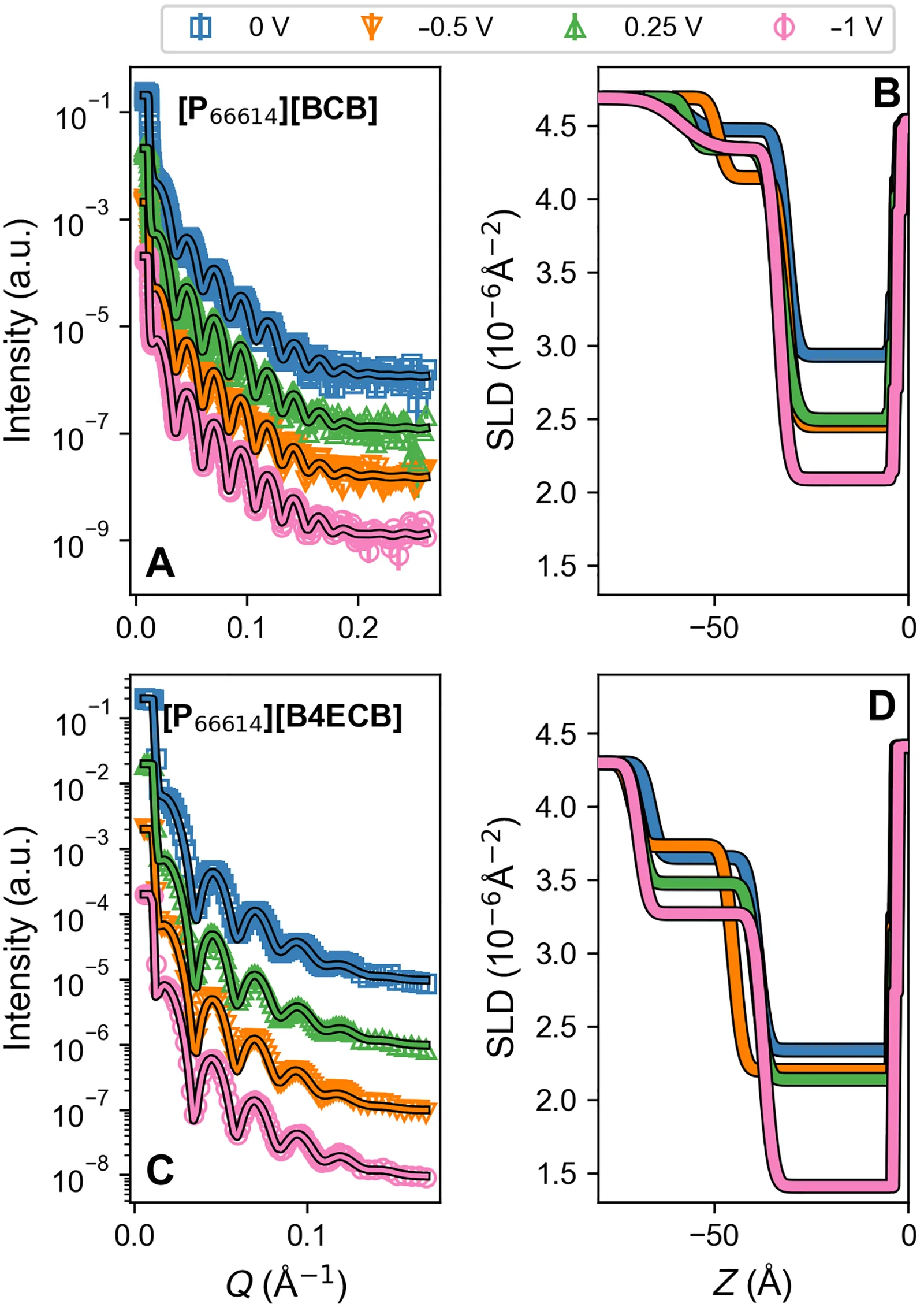
NR data and analysis of dispersed ILs. (**A** and **C**) Neutron reflectivity (NR) data (markers) and two-layer model fits (colored lines) taken on PLATYPUS (ANSTO) and SuperADAM (ILL), for the respective ILs in *d*_6_-propylene carbonate as a function of potential, where the order of the legend corresponds with the sequential ordering of applied potentials, where blue is 0 V, orange is −0.5 V, green is 0.25 V, and pink is −1 V; (**B** and **D**) scattering length density (SLD) profiles for the respective ILs arising from the two-layer model fits (note the common *x*/*y*-axis scaling). The legend applies to all plots and the order (sinistrodextral) corresponds to the experimental sequence of applied potentials.

The neutron reflectivity data in Fig. 4 demonstrate remarkable surface affinity and electroresponsivity at the gold solution interface and allow us to unambiguously characterize the EDL structure. This is observed as Kiessig fringes in the reflectivity data in Fig. 4 (A and C) ([BCB] and [B4ECB], respectively) that arise from an interfacial layer whose scattering length density (SLD) is different to that of bulk solution and the gold surface (which are matched to each other). Modeling of these Kiessig fringes using a differential evolution approach produces an SLD profile describing the interfacial layering away from the gold surface and into the bulk (toward −*Z* in Fig. 4, B and D). The context for this is formed by previous NR data available for the conventional reference borate systems of [BOB] and [BMB] (*21*, *22*). In all cases, two layers are required to fit the data. The near-surface layer is well defined and corresponds with the dimensions of the extended cation or ion pair. For [B4ECB], the layer is thicker, reflecting the larger anion. The slightly lower SLDs reflect both the higher hydrocarbon content and also a slightly increased adsorption, but once again, the layer thickness is consistent with the ion dimensions. The second layer has an SLD much closer to the bulk value, suggesting a much higher solvent content and/or patchiness.

#### Electro-responsive interfacial properties

Major changes in the structure of this interfacial layer are observed in response to an electric potential, and described as the electroresponsivity of the IL. Beginning at the open circuit potential (OCP), the low SLD value of the near surface indicates both a high surface affinity and a high cation content, because the cation has by far the lowest SLD value. This surface activity is greater than observed for this cation with the conventional, nondelocalized reference anions (*21*, *24*). In addition, the electroresponsivity of the layers is very large, as witnessed by the change in SLD value with applied potential (see comparison with literature in fig. S24). Both systems display the same electro-conditioning behavior seen earlier: This is a relatively recent observation, revealed in the SLD profile upon exposure to small voltages (which is largely independent of the sign) (*53*). Somewhat counterintuitively, because of short screening distances combined with very slow surface rearrangement kinetics, an induced bilayer structure such as this can partially resist a small reversal in surface charge, while still maintaining capacitive balance (*7*). The electro-conditioning thus seems to indicate that the electric field increases the surface excess. However, above a threshold negative potential, there is a pronounced drop in the fitted SLD profiles, indicative of a substantial enhancement in the cation content of the layer, which may be due to depletion of anions, enrichment of cations, or both. The overall dimensions do not change, but in the case of [B4ECB] anions, this cation enrichment is seen for both the inner and outer surface layers.

Rather than the conventional chequerboard structures for spherical or near spherical ILs, the well-defined interfacial layers are characteristic of self-assembly induced layers, which may either crowd or even overscreen the surface charge. The presence of the outer surface layers (with thickness characteristic of cation dimensions) supports this picture, because they imply the presence of intercalation of the catechol anions into the self-assembly induced structures of the primary layer. The second layer is thus required for overall neutralization of the surface charge.

### DFT calculations

**Fig. 5.**
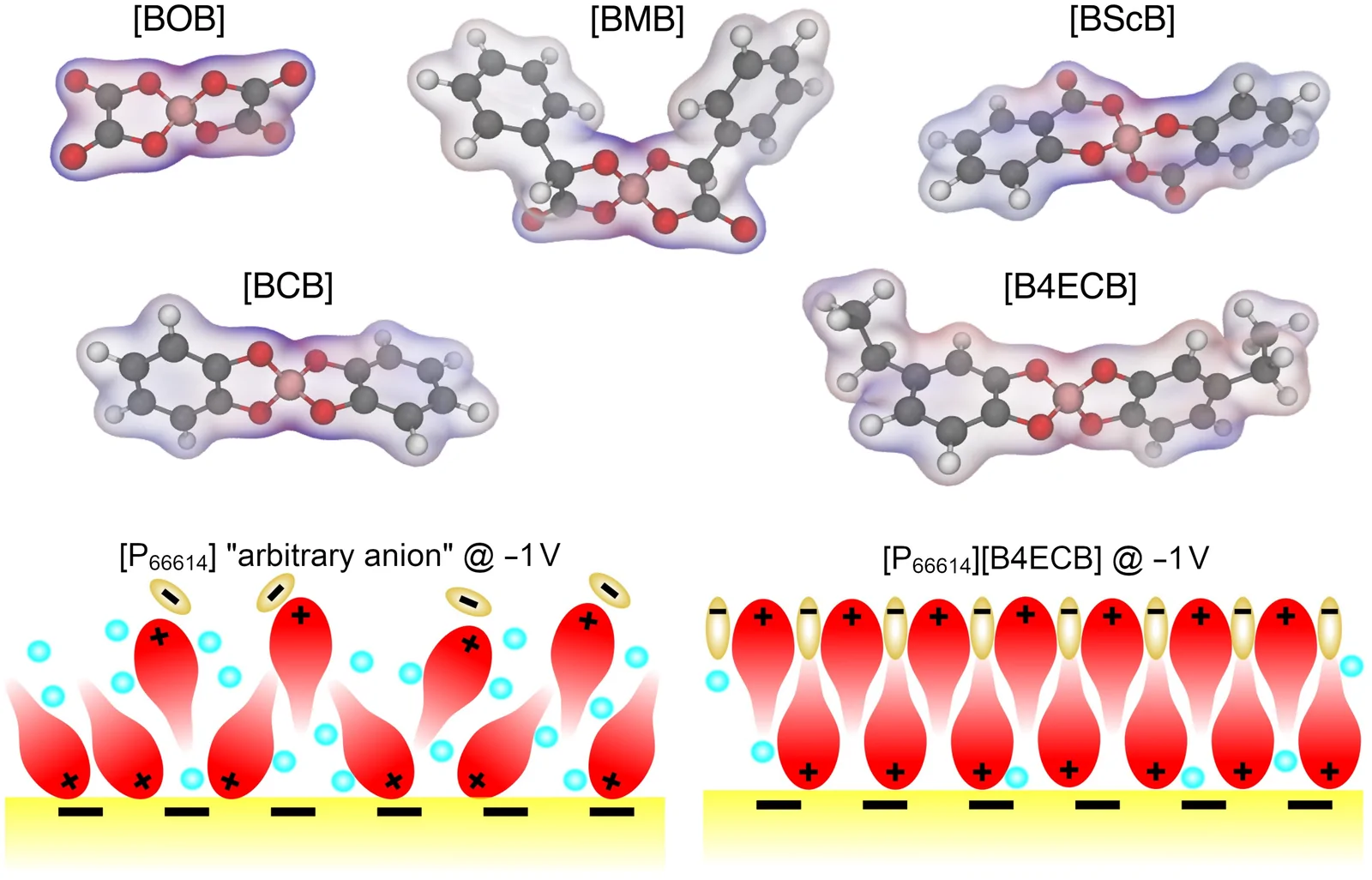
DFT analysis and surface structuring. Electrostatic potential surfaces for the three reference orthoborates (first row) and the two catecholatoborates (second row) where C atoms are gray, O atoms are red, B atoms are pink, blue isosurface coverage denotes regions of negative charge, red isosurface denotes positive charge, and white isosurface is electroneutral. Cartoons demonstrating the degree of interfacial ordering for [P_66614_] paired with a reference orthoborate (lower left) and for [B4ECB] (lower right) at an applied surface potential of −1 V, stabilized by anion intercalation in the self-assembly structures. Note that due to the 3D rendering (i.e., smoothing, shadowing, ambient occlusion, and, hence, overall raytracing) effects, electrostatic potential surface maps such as these should always be interpreted as semiquantitative despite plotting to the same color scale as that found in fig. S23; definitive lists of calculated charges per site from DFT are provided in the Supplementary Materials.

Ab initio calculations focused on the electronics of the orthoborate anions allow the rationalization of these multimodal experimental observations (refer to the Supplementary Materials for full details, and the electrostatic potential anion surface maps in Fig. 5). Namely, stark differences in anion electron density distribution are evident from the calculated anisotropic polarizabilities (Δα in table S9), alongside the response of the calculated anion Mulliken charges to a proximal +1 point charge placed 5 Å from the B center as demonstrated in the 2D electrostatic potential plots in fig. S23, and also the per-anion root mean square Mulliken charge deviation from these charge placement experiments in table S9. A physical basis is therefore provided for the “net” polarizability; the trends in all of these parameters clearly implicate intra-anion charge mobility for the catecholate systems, where the magnitudes follow the same [BOB] < [BMB] < [BScB] < [BCB] < [B4ECB] trend series from experimental SWAXS. Clearly, the π system is contributing beyond simple inductive effects and gains the ability to form an anion-spanning resonance system, expanding the chromophore region as the electrons become less localized. [BOB] is the most “ideal” anion here, in that it is the most geometrically compact, and closest to spherical, without delocalization. Appending two phenyl groups to [BOB] to create [BMB] decreases the polarity, and slightly increases the aggregate radius and length, but again not in excess of the ion dimensions. Self-assembly effects begin when the five-membered B-O-C ring of [BOB] and [BMB] expands to six-membered for [BScB], and the aggregates exceed ion pair dimensions. The distribution of charge across the orthoborate species is clearly of paramount importance, as [BMB] and [BScB] are very closely chemically related, but the phenyl ring is one step closer to the B center in the case of [BScB]. Thus, while the catecholates revert to a five-membered B-O-C ring structure, the conjugated ring system is now capable of integrating with the B center to form a much larger-scale delocalization, allowing the charge to spread further from B through electron induction, modifying overall polarizability.

The combination of SANS and NR electroresponsivity data shows that these nonaqueous catecholate IL systems function analogously to aqueous wormlike micelle assembly (*26*), where packing and curvature are controlled via the addition of simple salts or hydrotropes to modulate electrostatic screening effects (*54*–*59*). [BCB] and especially [B4ECB] form particularly thick near-surface layers with lower SLD values at OCP; the greater interfacial penetration of more polarizable anions stabilizes these high-density cation assemblies (cf. table S3). For [B4ECB], this strongly ordered first layer always exceeds 30 Å in depth, and a second layer is also obvious upon applied potential. Thus, while this adaptive self-assembly property is specifically revealed here for orthoborate anions (Fig. 5, bottom), it is by no means limited to them. We speculate that electronic resonance within any suitable anion could lead to the ability to redistribute its charge to satisfy the constraints of both solvophobicity and Coulombic interactions.

This extended delocalization of catecholate anions has potential application in solvent design (*60*). Greater polarizability, shown here as responsiveness to electrostatic environment, has potential in affecting solubility, with this responsiveness allowing a broader range of solvation interactions and hence a wider solubility window (*61*). Such interactions may themselves be extended to focus on species along a reaction coordinate, with these ILs offering a new way to consider reaction control (*62*).

Two ILs are introduced, based on the chelated orthoborate anions [BCB] and [B4ECB] paired with the [P_66614_] cation. While they share the same formal ionic borate center as more studied and conventional orthoborates such as [BOB], [BMB], and [BScB], they display remarkable differences. Detailed interrogation has elucidated (i) the neat liquid bulk structure using SWAXS, (ii) the bulk solution structure in a model nonaqueous polar aprotic solvent (propylene carbonate) using SANS, (iii) the solid-liquid solution structure at a gold electrode interface using NR, and, crucially, (iv) the electronic structure of the anions using DFT.

The catechol-based ILs showed the greatest nanodomain segregation in bulk SWAXS measurements, in keeping with the *L*_3_-like phase known for autoassemblies of ILs. In solution, catecholate anions cause unexpected increases in the size and definition of the self-assembly structures. This is the case for both the bulk structures measured by SANS and the interfacial near-surface layer observed by NR. We conclude that the emergent properties, particularly the electron distribution from DFT and the consequently marked effects upon experimental self-assembly behavior, are a direct product of resonance across the catecholates; this is forbidden in the conventional orthoborates.

The enhanced catecholate polarizability from this modified electron distribution imbues them with an adaptive ordering behavior. In self-assembly terms, catecholates can better intercalate to interfaces and aggregates and, through an optimization of both Coulombic and solvophobic interactions, can modify the local curvature of bulk aggregates and improve the packing density on an electrode surface. We would see this as a direct nonaqueous analogy to the use of salicylate in aqueous solutions of cationic surfactants, where replacing nonresonant anions with salicylate changes the micelle morphology from spherical to wormlike.

In IL systems, the self-assembly drivers are somewhat different, because hydrophobicity (an entropy driven phenomenon) is absent. Nonetheless, the self-assembly is driven by the balance of Coulombic and solvophobic interactions. This work thus provides a new axis in the control of IL structure and properties in both bulk (solution) and at interfaces. Earlier papers have shown how incremental improvements in IL boundary film ordering can substantially improve lubrication properties. The unprecedented self-assembly control afforded by the delocalization mechanism described here would be expected to substantially enhance this effect, and is consequently of significant interest beyond the IL community, providing a simple design criterion for lubricants, cosmetics, drug delivery systems, and battery and capacitor electrolytes. In particular, the ability to affect the EDL has profound implications for the control of electro-active lubrication and specific capacitance. Our findings therefore prove that the altered electron distribution arising from the bis(catecholato) π resonance allows for more efficient penetration of the anion into the charged [P_66614_] self-assemblies. Taking this proof one step further, and inserting further e-density into the π system by appending ethyl groups to [BCB] to form [B4ECB], the magnitude of the effect increases. Thus, the adaptability of the anions allows the twin imperatives of Coulombic and solvophobic self-assembly to be simultaneously accommodated.

## MATERIALS AND METHODS

The orthoborate salts sodium bis(catecholato)borate Na[BCB] and sodium bis(4-ethylcatecholato)borate Na[B4ECB] were prepared by adapting literature procedures for the preparation of acid-chelated orthoborates (fig. S1) (*63*). Fine details for these procedures, and full characterization of the prepared materials, are provided in the Supplementary Materials. DFT calculations were performed of the five orthoborate anions using Gaussian, with both geometry and energy calculated at the M06-2X/6–31++G** level of theory; only the *S*,*S*,*S* configuration of [BMB] was simulated. Polarizability tensors were calculated, and a charge delocalization experiment was performed by placing a +1 point charge 5 Å from the B center. SANS measurements of the two bis(catecholato)borate ILs and the three reference orthoborate ILs were made using the BILBY SANS instrument at ANSTO in Sydney, Australia (*64*, *65*). SWAXS measurements of neat ILs were taken using a Xenocs Xeuss 3.0 system with a single Dectris Eiger 2R detector in WAXS mode, with access provided by the ACNS under proposal P16734. NR measurements of [P_66614_][BCB] were performed at the PLATYPUS time-of-flight neutron reflectometer (*66*), also at ANSTO, whereas for [P_66614_][B4ECB], similar measurements were performed on the SuperADAM reflectometer at the Institut Laue-Langevin, Grenoble, France (*67*). Further Supplementary Materials are provided in support of this article: full synthetic details and characterization of prepared compounds, and minutiae of DFT, SAXS, SANS, and NR analyses.

## Supplementary Materials

### This PDF file includes:

Supplementary Text Figs. S1 to S24 Tables S1 to S9 References
