# Supplementary material for: Adaptive ionic moieties for electrolyte structural design
Source: Sci Adv. 2026 Sep 25;12(39):eaeh0276. doi: 10.1126/sciadv.aeh0276 (PMC13614367; doi:10.1126/sciadv.aeh0276)
Supplement: Supplementary file 1 [file sciadv.aeh0276_sm.pdf]

Science Advances
AAAS

Supplementary Materials for

Adaptive ionic moieties for electrolyte structural design

Oliver S. Hammond *et al.*

Corresponding author: Oliver S. Hammond, oliver.hammond@ess.eu;
Anja-Verena Mudring, anja-verena.mudring@bce.au.dk; Oleg N. Antzutkin, oleg.antzutkin@ltu.se;
Sergei Glavatskih, segla@kth.se; Mark W. Rutland, mark@kth.se

Sci. Adv. **12**, eaeh0276 (2026)
DOI: 10.1126/sciadv.aeh0276

This PDF file includes:

Supplementary Text
Figs. S1 to S24
Tables S1 to S9
References

Synthesis and characterization of bis(catecholato)borate ionic liquids

Instrumentation

^{1}H, ^{11}B ^{13}C and ^{31}P nuclear magnetic resonance (NMR) spectra were recorded at room temperature in DMSO-d_6 on a Bruker Avance 500 MHz (Ettlingen, Germany) and a Bruker 400 MHz spectrometer equipped with a BBO probe (Karlsruhe, Germany). Chemical shifts are reported in delta (δ) units, expressed in parts per million (ppm). The following abbreviations are used for the observed multiplicities: *s* (singlet), *d* (doublet), *t* (triplet), *q* (quartet), *br* (broad), *m* (multiplet for unresolved lines). ^{1}H NMR chemical shifts were referenced to the residual solvent signal for DMSO (2.50 ppm) and ^{13}C NMR chemical shifts were referenced to the solvent signal of DMSO (39.52 ppm). Fourier Transform Infrared spectra (FT-IR) were collected on a Bruker Alpha-P ATR-spectrometer equipped with a diamond crystal (Bruker, Ettlingen, Germany) in attenuated total reflection configuration. The data evaluation was carried out with the program OPUS (Bruker, Ettlingen, Germany). Differential scanning calorimetry (DSC) was performed with a computer-controlled DSC3+ STARe System (Mettler Toledo, Stockholm, Sweden). Measurements were carried out at a heating rate of 5 °C/min in a sealed aluminium crucible with a nitrogen flow rate of 50 mL/min. The samples were placed in aluminium pans (in air) which were cold-sealed and punctured. The given temperature of glass transition corresponds to the midpoint. ThermoGravimetric Analyses (TGA) were performed with a TG 449 F3 Jupiter (Netzsch, Selb, Germany). Measurements were carried out in aluminium oxide crucibles with a heating rate of 10 °C/min and nitrogen as purge gas. Given temperature corresponds to a 5% onset.

General synthesis procedure for ILs

All the starting materials (trihexyl(tetradecyl)phosphonium bromide ([P_{66614}][Br]), boric acid, sodium hydroxide, 4-ethylcatechol, and ethyl acetate) were purchased from Sigma-Aldrich (Steinheim, Germany). The novel bis(catecholato)borate salts were prepared by adapting a procedure found in the literature (Figure S1).*(63)* Namely, trihexyl(tetradecyl)phosphonium bromide (10 mmol, 1.0 eq), was combined with the respective sodium bis(catecholato)borate species (1.2 eq) and water (20 mL) in a flask with stirring, and left overnight at room temperature before extracting three times with ethyl acetate. The organic phases were combined and dried over anhydrous magnesium sulphate. After filtration over cotton, the solvent was removed in vacuo to yield the final product.

Trihexyl(tetradecyl)phosphonium bis(catecholato)borate) [BCB]

Pale brown solid. ^{1}H-NMR (400 MHz, DMSO-d_6): 0.85-0.89 (*m*, 12H), 1.26-1.29 (*m*, 32H), 1.32-1.39 (*m*, 8H), 1.41-1.49, (*m*, 8H), 2.10-2.17, (*m*, 8H), 6.47 (*s*, 8H). ^{11}B-NMR (128 MHz, DMSO-d_6): 14.23. ^{13}C-NMR (101 MHz, DMSO-d_6): 13.8, 13.9, 17.4 (*d*, JC-P = 47.5 Hz), 20.5 (*d*, JC-P = 4.8 Hz), 21.8, 22.1, 28.1, 28.7, 28.7, 29.0, 29.0, 29.0, 29.1, 29.1, 29.7 (*d*, JC-P = 15.3 Hz), 30.4, 31.3, 107.5, 117.2, 151.6. ^{31}P-NMR (162 MHz, DMSO-d_6): 33.65. FTIR (ν/cm^{-1}): 3050, 2954, 2924, 2853, 1601, 1571, 1484, 1414, 1373, 1321, 1229, 1146, 1095, 1055, 1005, 942, 917, 906, 865, 797, 730, 700, 648, 553, 433, 408

Trihexyl(tetradecyl)phosphonium bis(4-ethylcatecholato)borate) [B4ECB]

Dark brown oil. ^{1}H-NMR (500 MHz, DMSO-d_6): 0.84-0.88 (*m*, 12H), 1.09-1.14 (*m*, 6H), 1.25 (*m*, 16H), 1.26-1.30 (*m*, 16H), 1.34-1.40 (*m*, 8H), 1.41-1.47 (*m*, 8H), 2.10-2.16 (*m*, 8H), 2.39-2.45 (*m*, 4H), 6.28 (*dd*, JH-H = 8.0 Hz, JH-H = 2.0 Hz, 1H), 6.32 (*s*, 2H), 6.33 (*d*, JH-H = 6.0

Hz, 1H), 6.42 (*dd*, JH-H = 8.0 Hz, JH-H = 2.0 Hz, 1H), 6.57 (*d*, JH-H = 2.0 Hz, 1H), 6.61 (*d*, JH-H = 8.0 Hz, 1H). ^{13}C-NMR (126 MHz, DMSO-d_6): 13.8, 13.9, 15.9, 16.4, 17.4 (*d*, JP-C = 47.4 Hz), 17.5 (*d*, JP-C = 47.4 Hz), 20.5 (*d*, JP-C = 4.4 Hz), 20.5 (*d*, JP-C = 4.4 Hz), 21.8, 22.1, 27.5, 28.1, 28.4, 28.7, 28.7, 29.0, 29.0, 29.0, 29.1, 29.1, 29.7 (*d*, JP-C = 15.0 Hz), 30.0 (*d*, JP-C = 15.0 Hz), 30.4, 31.3, 106.7, 107.4, 115.1, 115.4, 115.7, 118.1, 132.5, 134.5, 143.1, 145.1, 149.6, 151.6. ^{31}P-NMR (203 MHz, DMSO-d_6): 33.65. FTIR (ν/cm^{-1}): 3211, 3026, 2957, 2924, 2854, 1602, 1489, 1447, 1413, 1376, 1318, 1253, 1225, 1141, 1093, 1049, 978, 929, 904, 854, 805, 719, 695, 635, 588, 498, 460, 435. T_{glass} = -49 °C; T_{decomp} = 270 °C.

Details of small/wide-angle X-ray scattering (SWAXS) experiments

Data collection

SWAXS measurements were performed using a Xenocs Xeuss 3.0 instrument, located at the Australian Centre for Neutron Scattering (access provided under beamtime allocation P16734). The instrument uses Cu-Kα radiation and a single Dectris Eiger2 SAXS detector placed directly in front of the sample, to achieve a wide Q-range of 0.09 – 3.88 Å^{-1}. Neat ILs were vacuum-dried and hermetically sealed into 1.5 mm quartz glass capillaries, wherein they were generally measured for an exposure time of 1 h, with a Peltier stage regulated to 25 °C at the sample position. No long equilibration time effects were observed for this family of ILs.*(68)* The bis(catecholato) ILs were measured at various temperatures. In particular, the [BCB] temperature scans involved changing the temperature at a rate of 5°C per minute, then equilibrating at the new temperature for 1 minute, prior to 5 minutes of X-ray exposure. The sample was held at the lowest temperature for approximately 90 minutes prior to heating. Data were background-subtracted and normalized to an absolute scale prior to further processing. Peak integrals are provided in Table S1.

Assessing thermotropic behavior in [P_{66614}][BCB]

The SWAXS temperature scan data showing thermotropic behavior (lamella formation and crystallization) for [P_{66614}][BCB] are shown in Figure S16. A lamellar structure was fitted to the lower-Q data below ca. <1 Å^{-1}, with a repeat distance of 35.155 ± 0.122 Å from Gaussian peak fitting of the 4 first features. Above ca. 0.8 Å^{-1}, the peaks in the 15°C sample likely correspond to a separate phase, with partial crystallization and the ensuing packing of ie. hydrocarbons. The nonlinear background contributions in the low temperature sample match well with the positioning of the broad fluid isotropic phase peaks, indicating incomplete phase transition. This interpretation fits very closely with the DSC scans: When the heating rate is slow (1K/min) (Figure S14), there is one sharp peak upon heating at 47.3°C corresponding to melting, and one sharp peak upon cooling at 8.0°C corresponding to a crystallization event. The difference of temperature between the two processes indicates supercooling behavior. However, when the sample is measured with a heating rate of 5K/min a different behavior is observed (see Figure S13). Upon the first heating cycle, the compound starts to melt at 29.8°C directly followed by a second event at 43.5°C into the isotropic liquid phase. During the first cooling, there is a weak transition at 5.8°C, which is a crystallization from isotropic liquid to liquid crystal phase where the liquid crystal structure is kinetically trapped. Upon the second heating, two cold crystallizations can be observed, firstly at -20.3°C which could correspond to a liquid crystalline phase to a solid phase, and secondly at -4.5°C which could be a solid-solid event. Then, the compound starts to melt at 29.8 °C directly followed by a second transition at 43.5 °C similar to the first heating. The second cooling cycle is identical to the first. The same type of thermal

history dependence has recently been well-described by Perkin and coworkers for related concentrated eutectic melts,*(69)* highlighting challenges and general good practice for studies of such systems with similarly challenging property sets.

Details of small-angle neutron scattering (SANS) experiments

Data collection and analysis

SANS measurements were performed using ANSTO's BILBY instrument, a small-angle reactor-based instrument with the capability for time-of-flight (ToF) operation. BILBY's small angle detector bank is complemented by several asymmetrically-arranged wide angle detector arrays. These measurements used velocity selection to obtain a nominally 4.5 Å (dQ/Q = 10%) neutron beam. The experimental Q-range was 0.0105 – 0.7212 $Å^{-1}$. The direct beam was measured, which alongside various background standards (H_2O, blocked beam, empty cell, etc.) allowed for reduction of data to an absolute scale using Mantid.*(70)* The open-source small-angle scattering software suite SasView was subsequently used both for model-based fitting and Indirect Fourier Transform analysis.*(71)* Example datasets of the anhydrous IL/solvent mixtures with cylinder + S(Q) fits are provided in Figure S17, and the same fits are shown alongside hydrated samples in Figure S18; the full set of fitting parameters for reproduction are given in Table S2.

Details of neutron reflectivity (NR) experiments

The NR cell

All NR measurements were performed using a custom-made electrochemical NR cell. We have previously reported the details of the cell design but have briefly described it here.*(21, 24)* A thin (200 Å) amorphous gold film was deposited on a 10 mm (50 × 50 mm) polished silicon (100) block (Crystran, Poole, United Kingdom) and used as the working electrode (WE) with a thin adhesion layer of titanium (Ti) on the silicon surface. Additionally, a short, insulated copper wire was adhered to one corner of the gold surface with conductive epoxy (CW400, Chemtronics) and cured in an oven at 120 °C for 20–30 min. After cooling, the gold surface was rinsed with absolute ethanol, dried with nitrogen, and exposed to UV/ozone for 10 min to remove any organic residues. A conductive glass (coated with fluorine-doped tin oxide, FTO, Sigma Aldrich) was used as the counter/reference electrode (CE/RE). For the measurements presented here, the cell only accommodates a two-electrode system. A PTFE gasket of 0.5 mm thickness was used to separate the CE/RE from the WE and to contain the IL solution. Before assembling the NR electrochemical cell, the conductive glass, gasket, and all other cell components were sonicated in 2% v/v Hellmanex® for 30 min at room temperature, followed by thorough rinsing with Milli-Q water and finally absolute ethanol, before drying with nitrogen. To minimize ambient water contamination, the cell was assembled inside a polyethylene glove bag under a dry argon atmosphere (R.H. < 10%). The IL solution was injected into the cell with the adapter using a glass Luer syringe. After mounting the cell on the beamline, electrical potentials were applied across the cell with a potentiostat (Metrohm Autolab, PGSTAT204). The gold-on-silicon block layers were characterized in air for their parameters (*i.e.*, SLDs, thickness and roughness), and used as references for the subsequent IL solution NR fitting (Figure 3).

NR methodology for [P_{66614}][BCB]

The NR measurements of P_{66614} BCB were performed at the PLATYPUS time-of-flight neutron reflectometer at ANSTO in Sydney, Australia.*(66)* To comprehensively cover the Q range from

the critical edge to the background (0.007 – 0.26 Å^{-1}), two incident angles of 0.65º and 3º were used. A constant dQ/Q resolution of approximately 5% was maintained across all measurements. To ensure a consistent beam footprint within the region covered by the gold-solution interface, slits were employed to precisely define the beam size. The NR measurements were performed with the electrochemical NR cell placed in a horizontal configuration. The PLATYPUS datasets from the two incident angles were combined to generate a unified NR profile.

NR methodology for [P_{66614}][B4ECB]

The NR measurements of P_{66614} B4ECB were performed on the SuperADAM reflectometer at the Institut Laue-Langevin (ILL), Grenoble, France.*(67)* The neutron specular reflectivity R (the ratio of intensity between the reflected and the incident beam) was measured as a function of Q. During the measurement, the wavelength was fixed at 5.21 Å, and the angle of incidence was varied, providing a Q_z-range of 0.006 – 0.17 Å^{-1}. For the solution NR measurement, the neutron beam was directed through the Si block, reflected from the gold-solution interface, before again traversing the block and exiting towards the detector. All the original scattering data were reduced according to the standard procedures of SuperADAM, using the program pySARed. These procedures include background subtraction, detector efficiency calibration, and over-illumination correction.*(23)*

Data processing and analysis

The reduced data were fitted with the software package GenX,*(72)* using a slab model in which the layer's thickness, roughness and SLD are constrained between super- and subphases.*(73)* The substrate parameters of the Ti and layers (*i.e.*, SLD, thickness, and roughness) were obtained from the reflectivity of the block measured in air and fixed for all subsequent potentials. Gold layer thickness and the bulk SLD were fitted for the first applied potential (*i.e.*, 0 V or OCP) and fixed for the subsequent potentials. A "micro-slabbing" approach was applied for the gold-IL ion interfacial region as reported in previous studies.*(25, 74)* This was done due to the gold layer roughness being comparable to the IL's molecular dimensions. This method creates a series of 1 Å thick slabs of 0 Å roughness, where the SLD of every micro-slab is determined based on the normal distribution of the volume fraction of gold and the 1st layer of IL ions. This "micro-slabbing" parameter is used instead of the gold-IL interfacial roughness.*(23)* Apart from these parameters, the SLD and thickness of the interfacial layers, as well as the roughness of the IL/bulk interface were allowed to vary for all the fitted datasets. For each simulation of different potentials, no pre-defined interfacial structures were assumed. Each fit began with the simplest model of a single layer, only increasing the number of layers if a physically plausible model was described with a decreased logarithmic figure of merit (FOM). The layer SLDs were constrained to be in the range of the possible component species. The SLD profiles were obtained from the best fits of the NR data, determined by the lowest logarithm of the FOM value with the minimum number of layers with physically plausible parameters.

Model selection

As described in the Methodology, the selection of each NR fit is based on the figure of merit (FOM) calculation and physically plausible model parameters. With the gold thickness, roughness and bulk SLD of consequent fits based on the first potential applied, to make thorough comparisons the number of fits multiplies. For this reason, Figure S21 shows the FOM values obtained for all 1- and 2-layer fits, allowing their comparison to help identify the best fit. Low

variance in the FOM was observed for these fits with the exception of the -1 V potential, where both ILs had a significant minimum FOM for the 2-layer fits. However, the B4ECB anion had a noticeable trend whereby the 2-layer fits based on a 2-layer OCP were consistently the lowest FOM, indicating a two-layer model of the system is the best description. The BCB anion was similarly represented, where a 2-layer 0 V results in the best model being a 2-layer fit of the system.

FOM is not the only indicator of a good fit, and so the physical parameters of the system must be considered as well. Therefore, the SLD plots and tabulated parameters are presented in both Figure S19 and Figure S20 for each series of fits. This shows that many of the 2-layer fits have well defined layers with low roughness while the 1-layer fits have higher roughness, resulting in very similar total thickness of the system. Our interpretation favors the 2-layer fit, as a more physically reasonable representation of the system than the 1-layer fits.

DFT calculation overview

Computational details

The five different orthoborate anions were constructed and geometry optimization was performed at the M06-2X/6-31++G** level of theory. Mulliken charges and polarizability tensors were calculated for the geometry-optimized structures. The charge response of the anions was further confirmed by recalculating, with a +1 point charge now placed 5 Å from the B center of the optimized geometries, perpendicular to the long axis of the anion, to determine changes in the Mulliken charges (*i.e.* redistribution and magnitude).

The skeletal structures of the anions are provided alongside the corresponding atom indices for reference in Figure S22. The calculated Mulliken charges of the optimized anions and also the differences observed when exposed to this +1 point charge, are provided in Table S3–Table S6. The calculated surface areas were used to normalize the anisotropic polarizability tensor, with these results provided in Table S7.

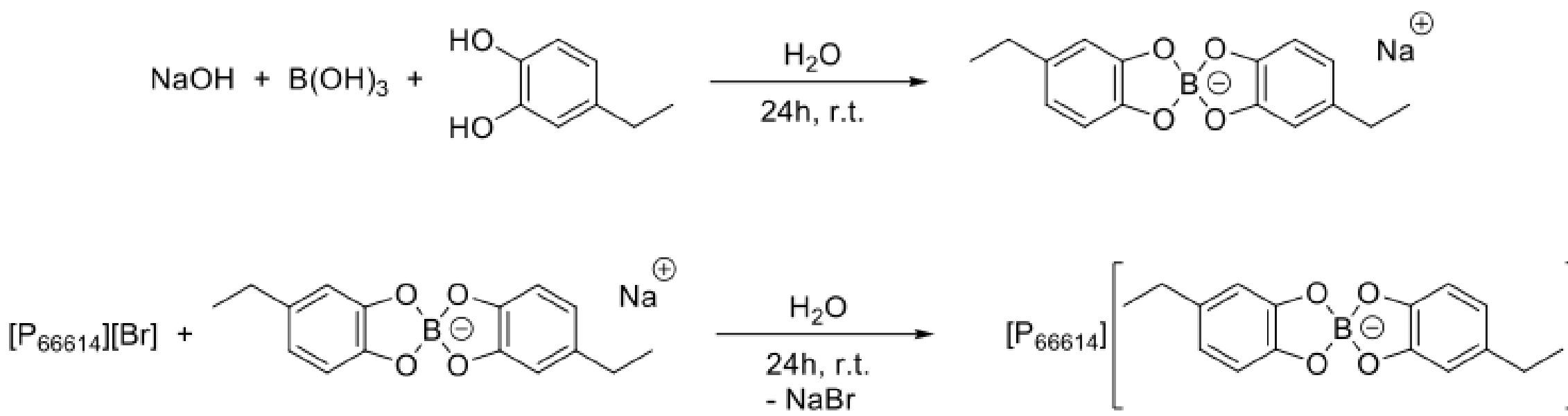

Fig. S1.

Example showing the synthesis pathway of the ionic liquid trihexyl(tetradecyl)phosphonium bis(4-ethylcatecholato)borate) [P_{66614}][B4ECB].

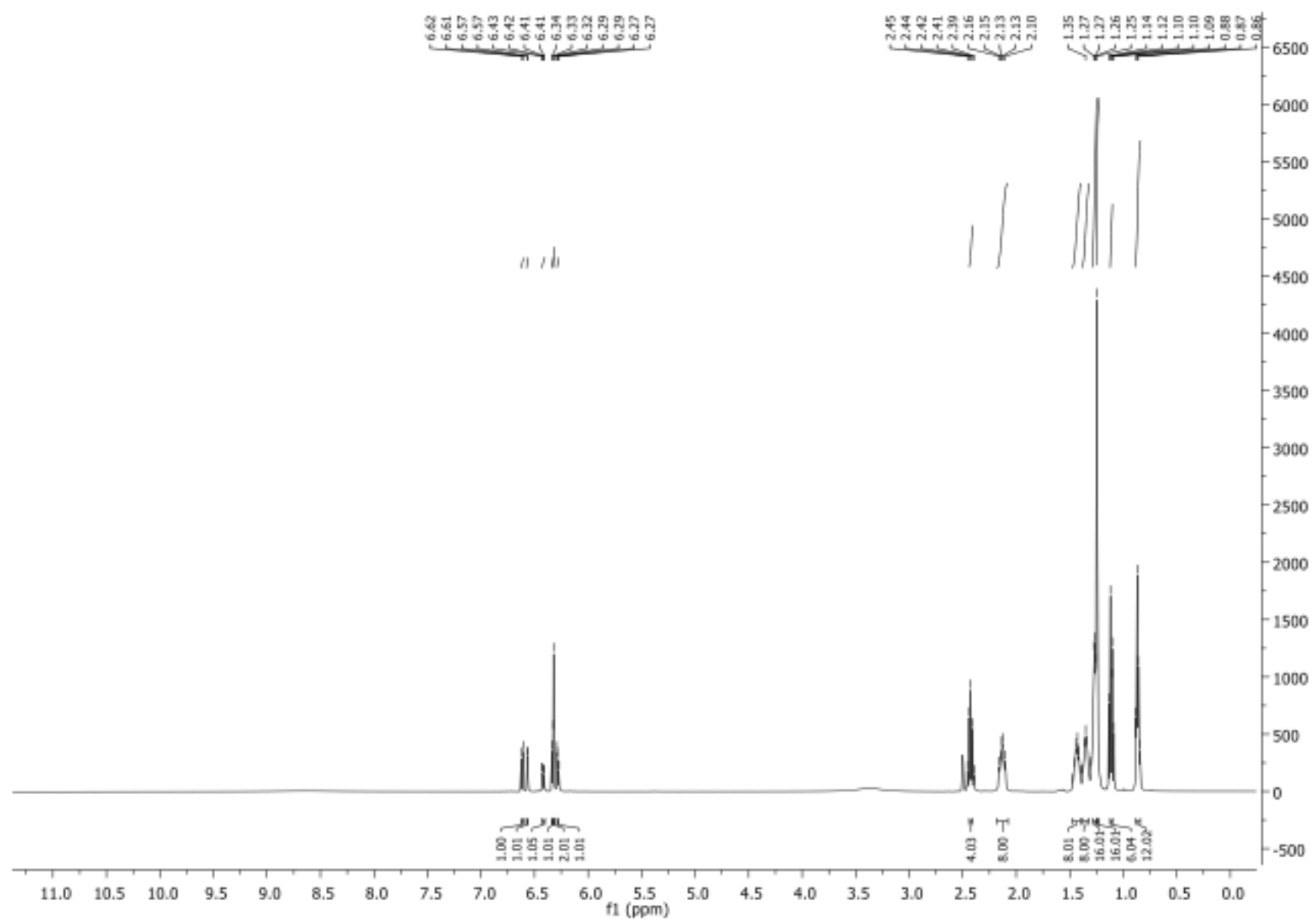

Fig. S2.

^{1}H-NMR spectrum (500 MHz, DMSO) of $[P_{66614}]$[B4ECB], including the full set of peak assignments and their respective integrals.

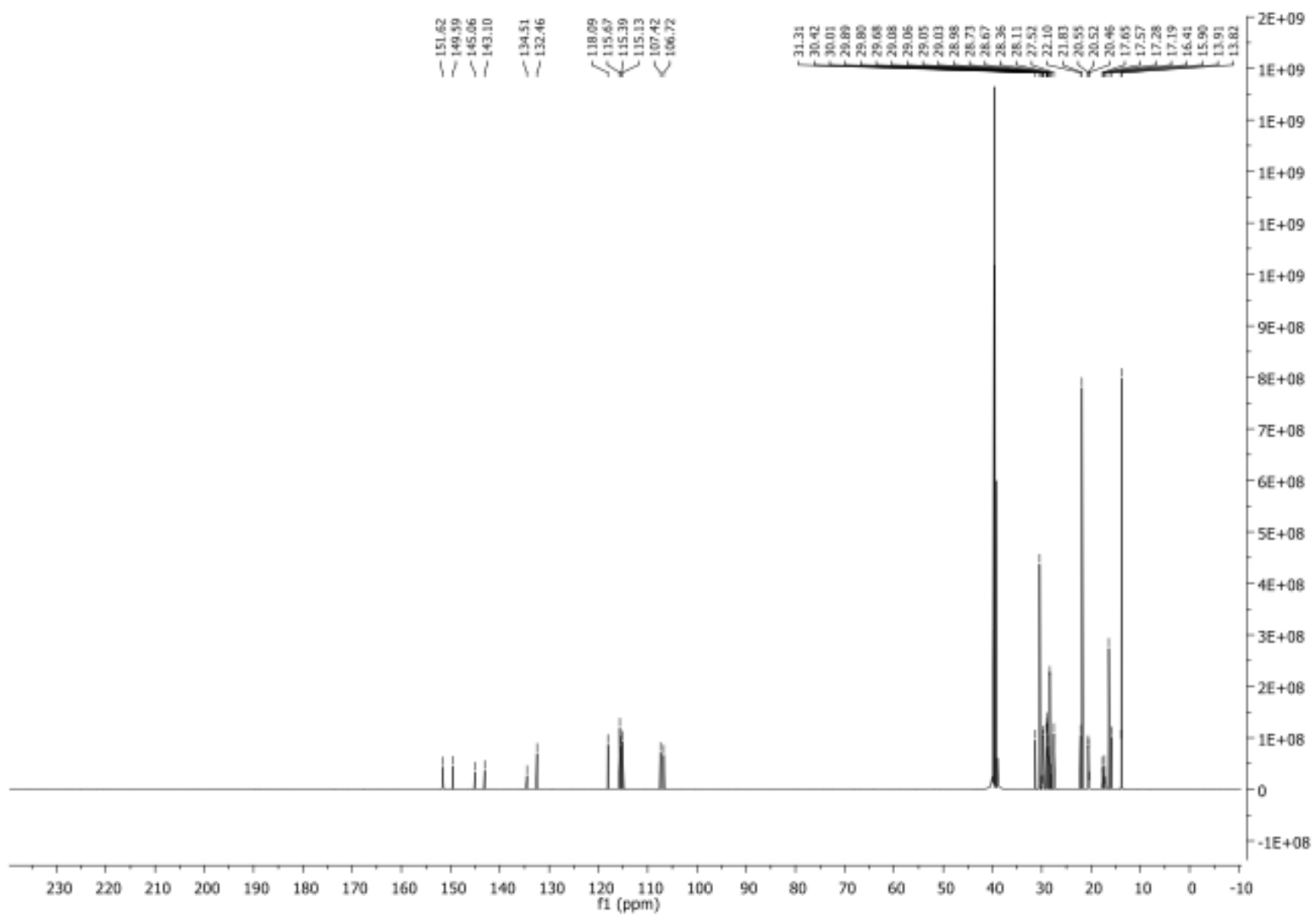

Fig. S3.
^{13}C-NMR spectrum (126 MHz, DMSO) of [P_{66614}][B4ECB], including the full set of peak assignments.

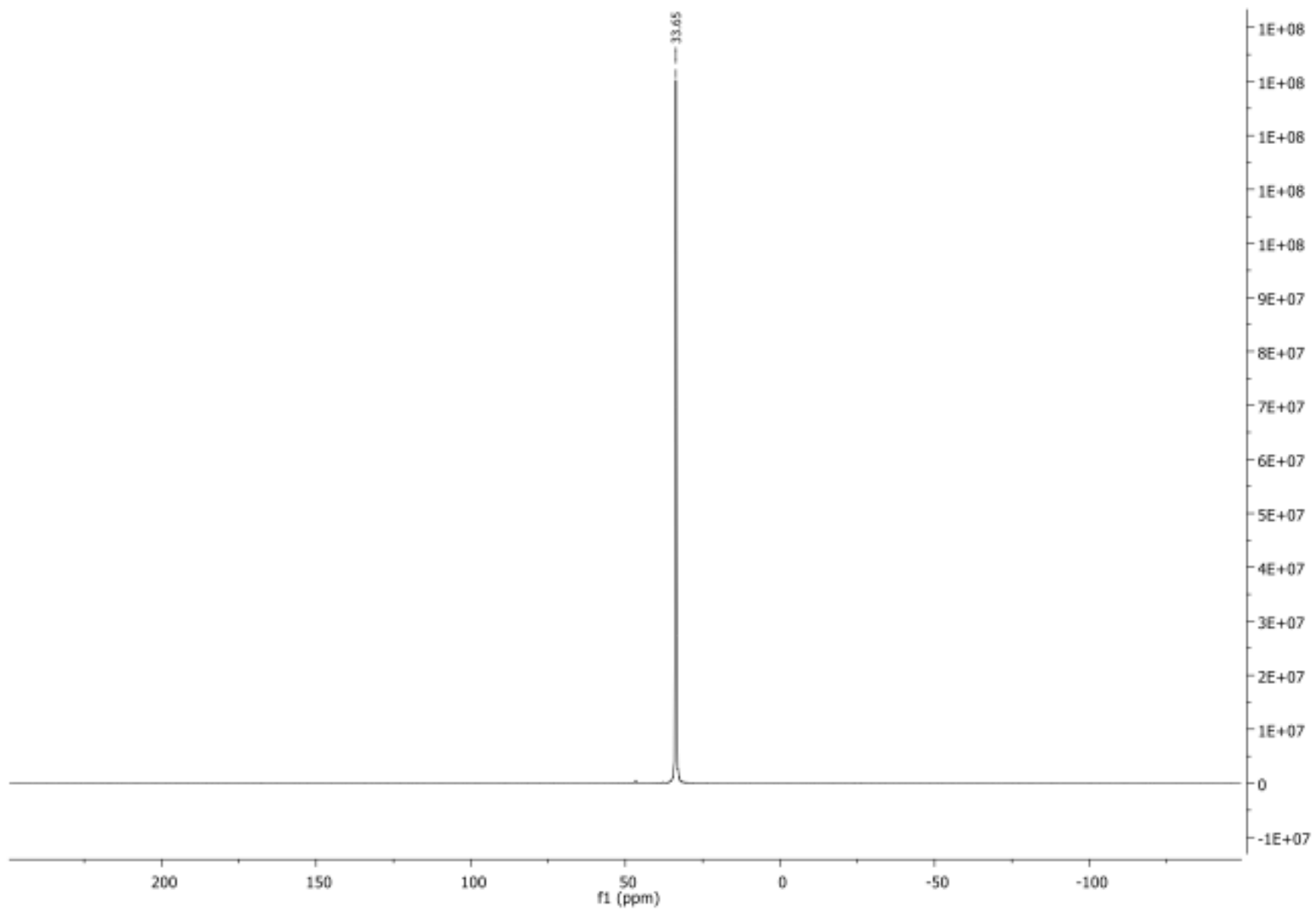

Fig. S4.
^{31}P-NMR spectrum (203 MHz, DMSO) of [P_{66614}][B4ECB], including the peak assignment.

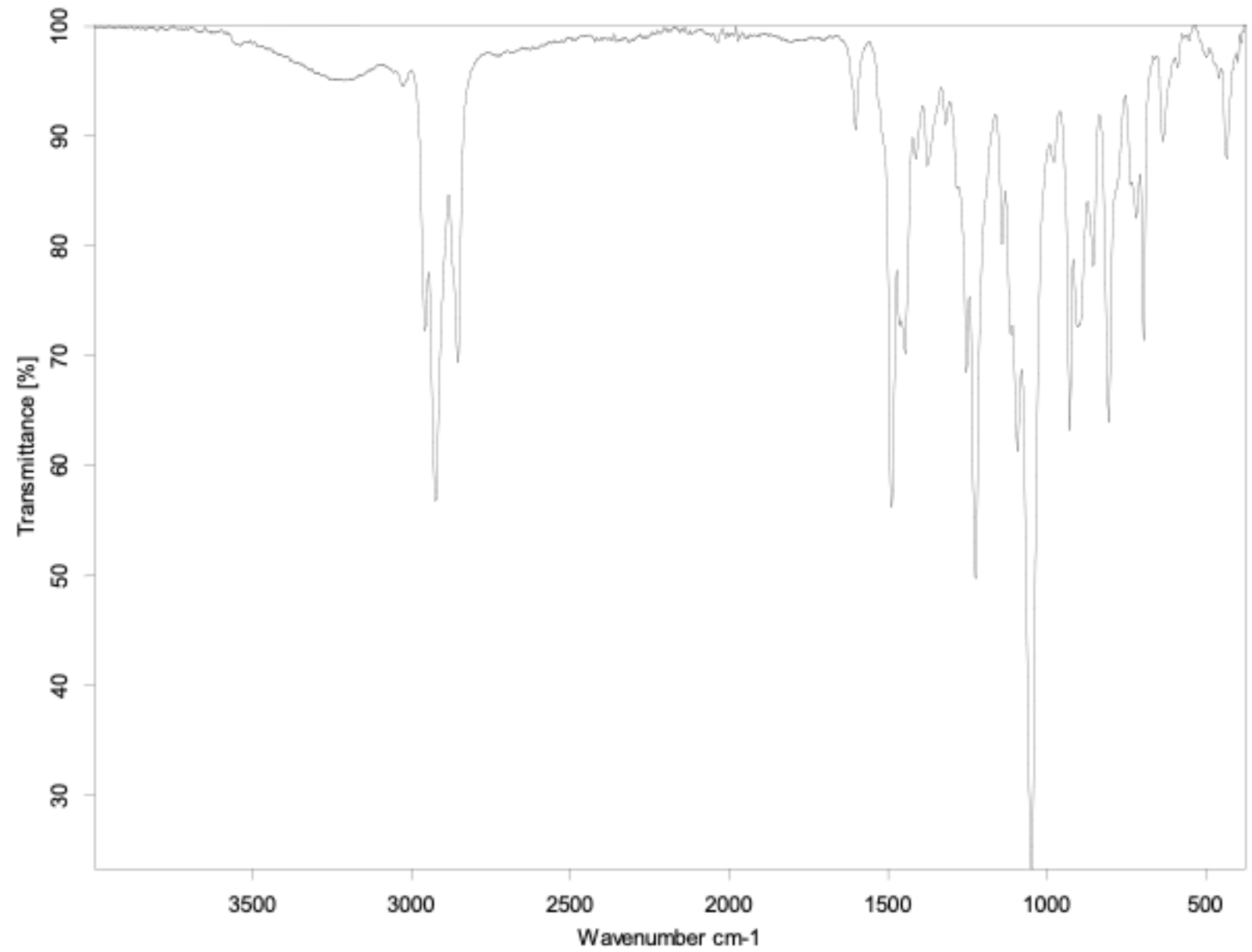

Fig. S5.

FTIR spectrum of [P_{66614}][B4ECB], collected using a Bruker Alpha-P ATR-spectrometer equipped with a diamond crystal.

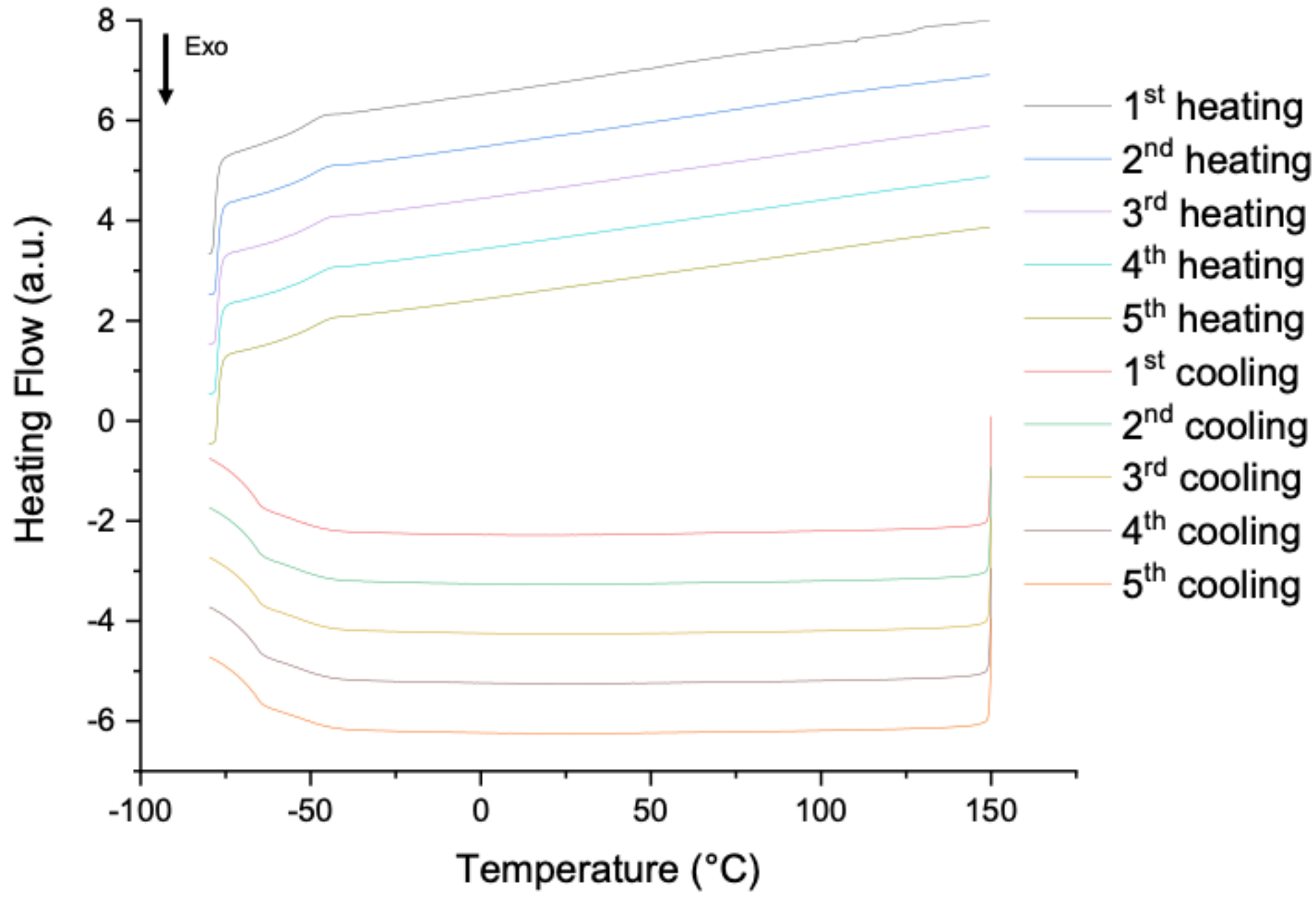

Fig. S6.

Results from Differential Scanning Calorimetry measurements of [P_{66614}][B4ECB] at a heating rate of 5K/min, measured using a Mettler Toledo DSC3+ STARe, with the sample cold-sealed in air in a sealed aluminium crucible under a nitrogen flow rate of 50 mL/min.

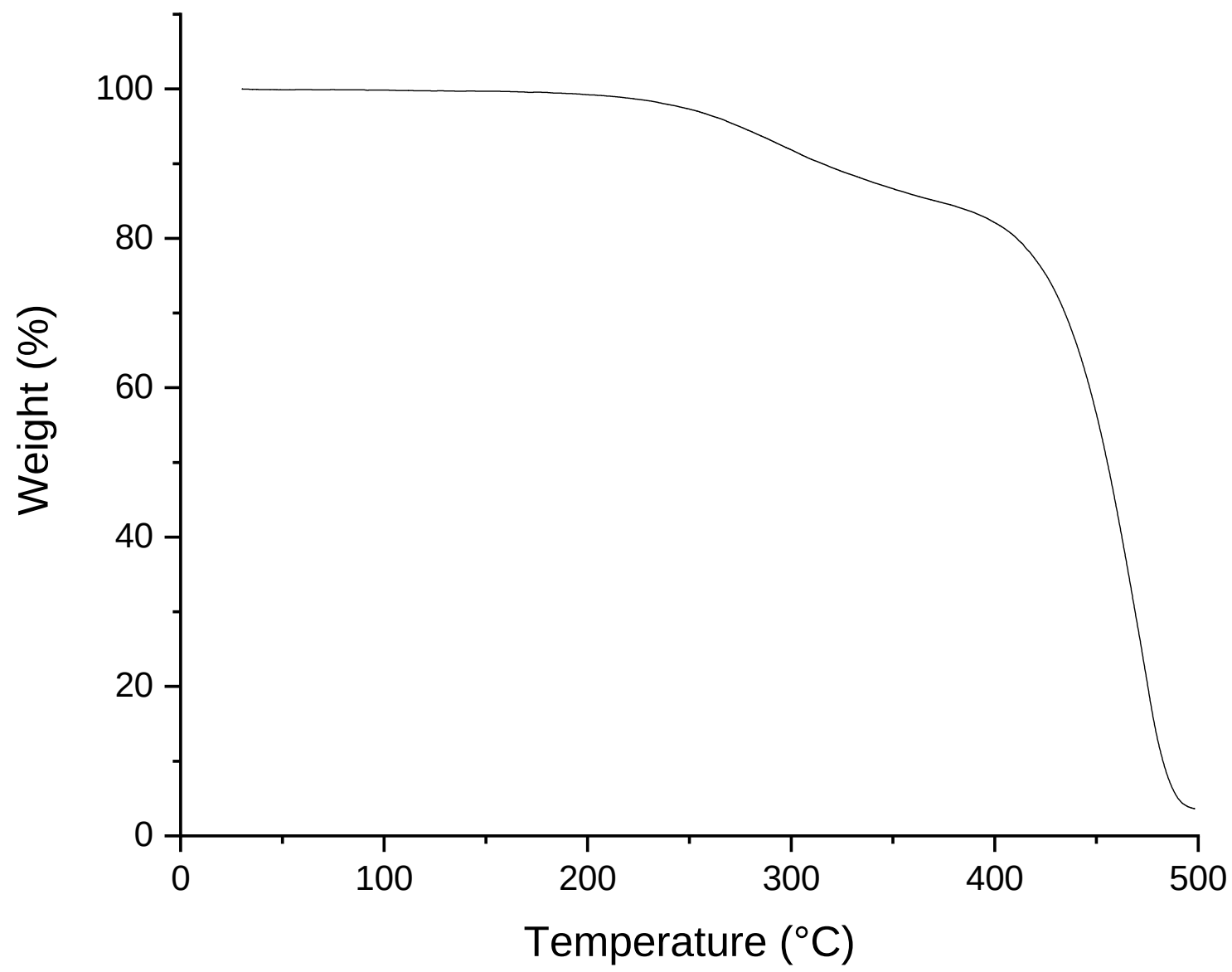

Fig. S7.

Results from thermogravimetric analysis of [P_{66614}][B4ECB], collected using a Netzsch TG 449 F3 Jupiter system with aluminium oxide crucibles, a heating rate of 10 °C/min and nitrogen as purge gas. A 5% onset was used for calculation of degradation temperatures.

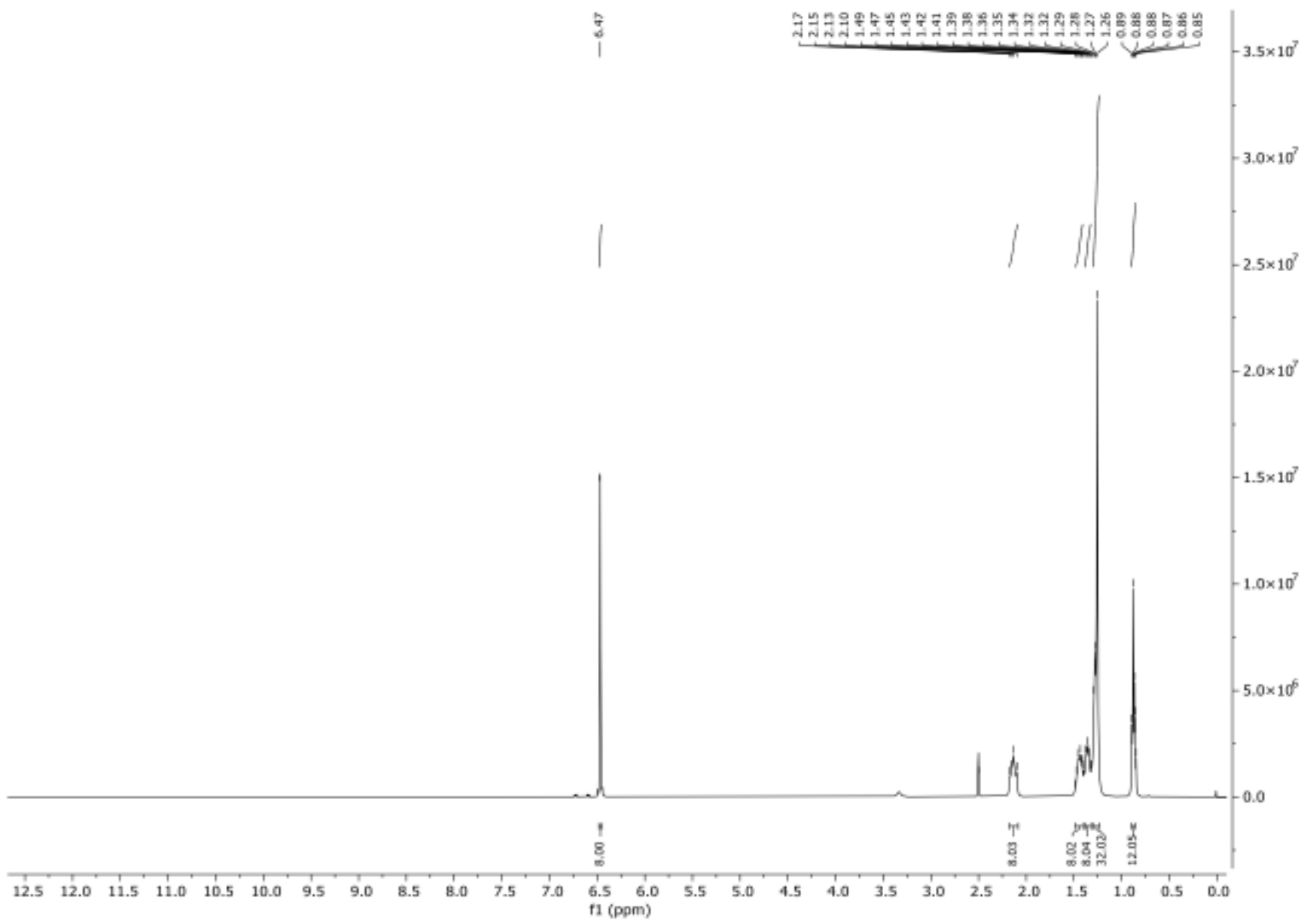

Fig. S8.

^{1}H-NMR spectrum (500 MHz, DMSO) of [P_{66614}][BCB], including the full set of peak assignments and their respective integrals.

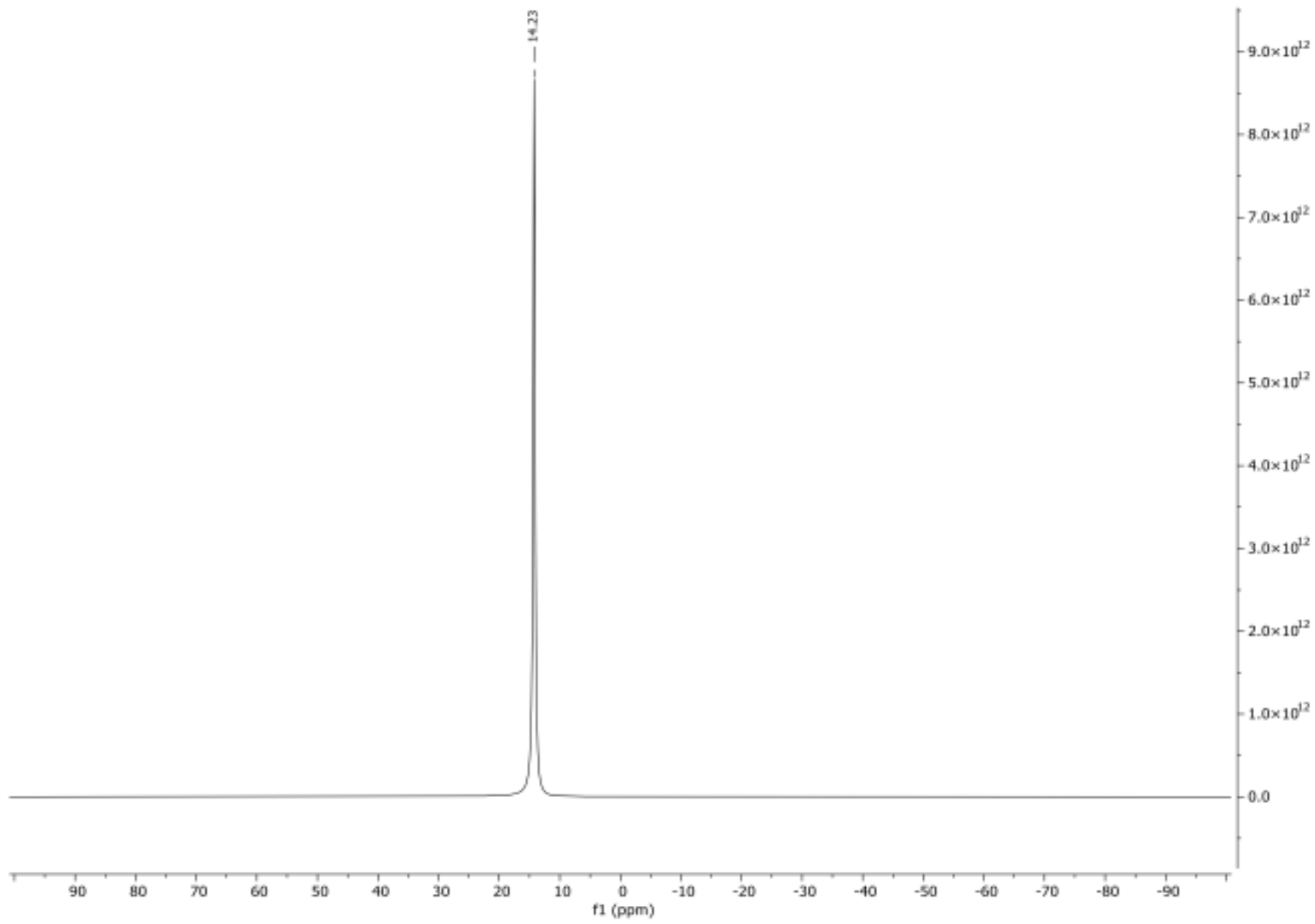

Fig. S9.
^{11}B-NMR spectrum (128 MHz, DMSO) of [P_{66614}][BCB], including the peak assignment.

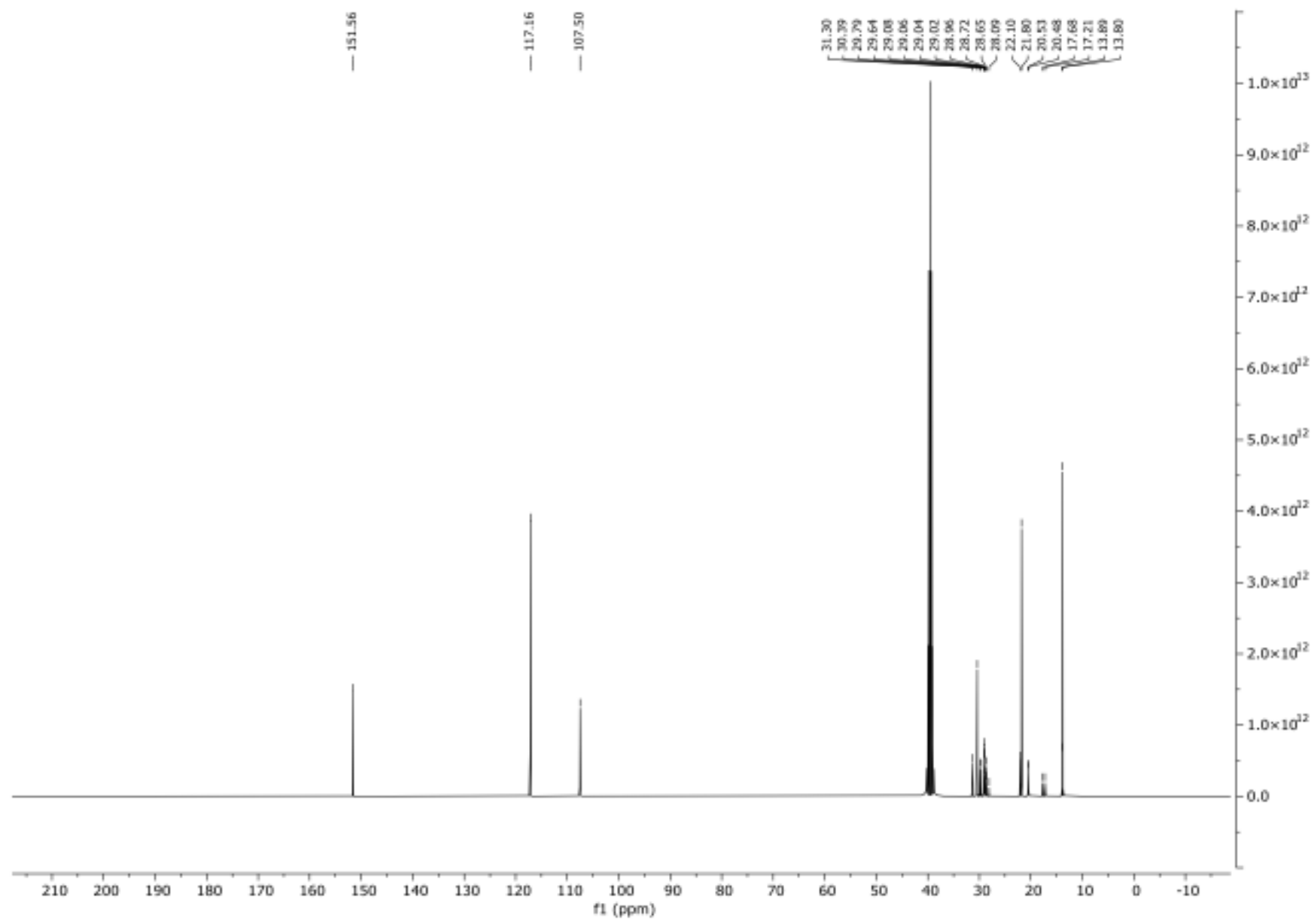

Fig. S10.
^{13}C-NMR spectrum (101 MHz, DMSO) of [P_{66614}][BCB], including the full set of peak assignments.

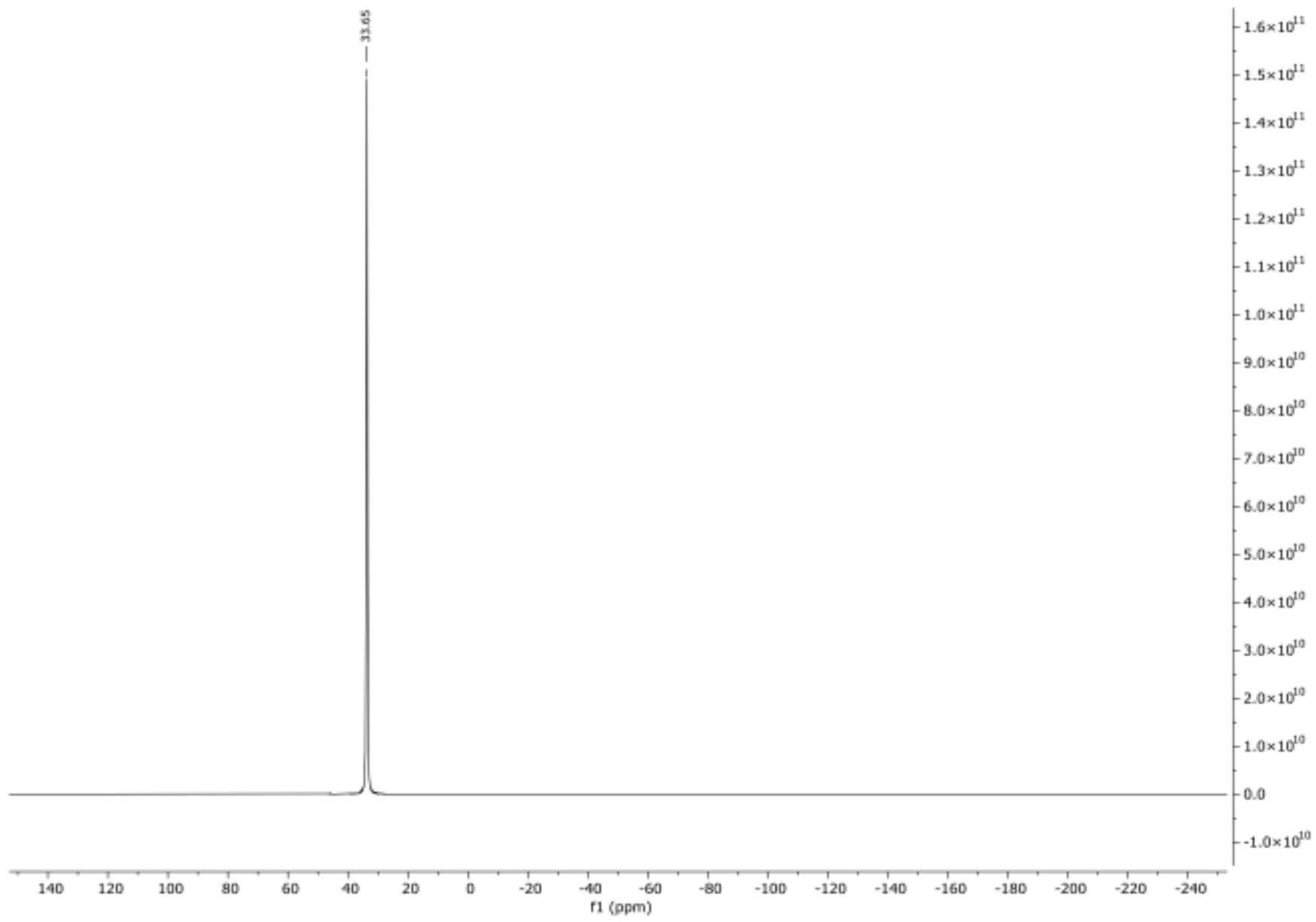

Fig. S11.

^{31}P-NMR spectrum (162 MHz, DMSO) of [P_{66614}][BCB], including the peak assignment.

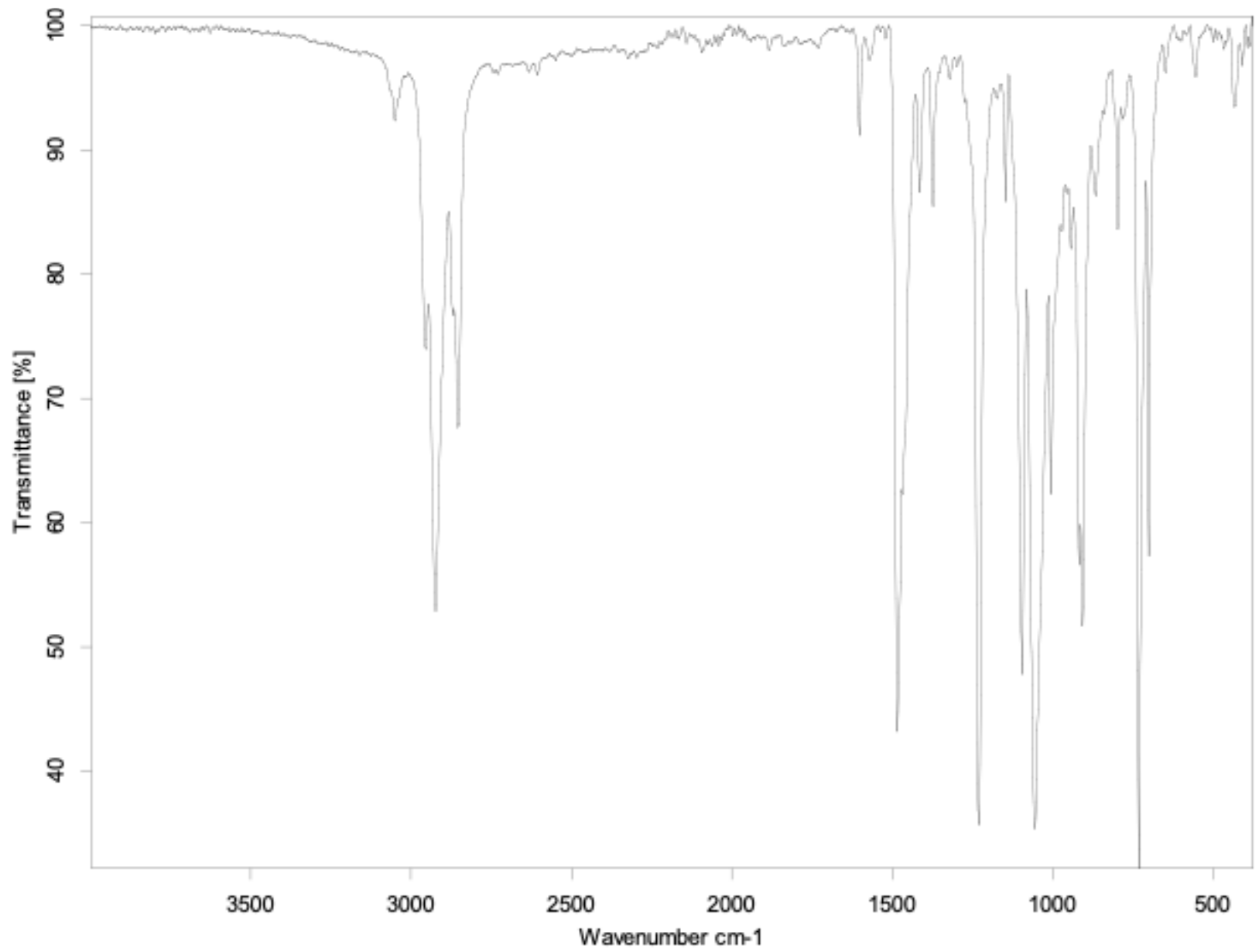

Fig. S12.
FTIR spectrum of [P_{66614}][BCB], collected using a Bruker Alpha-P ATR-spectrometer equipped with a diamond crystal.

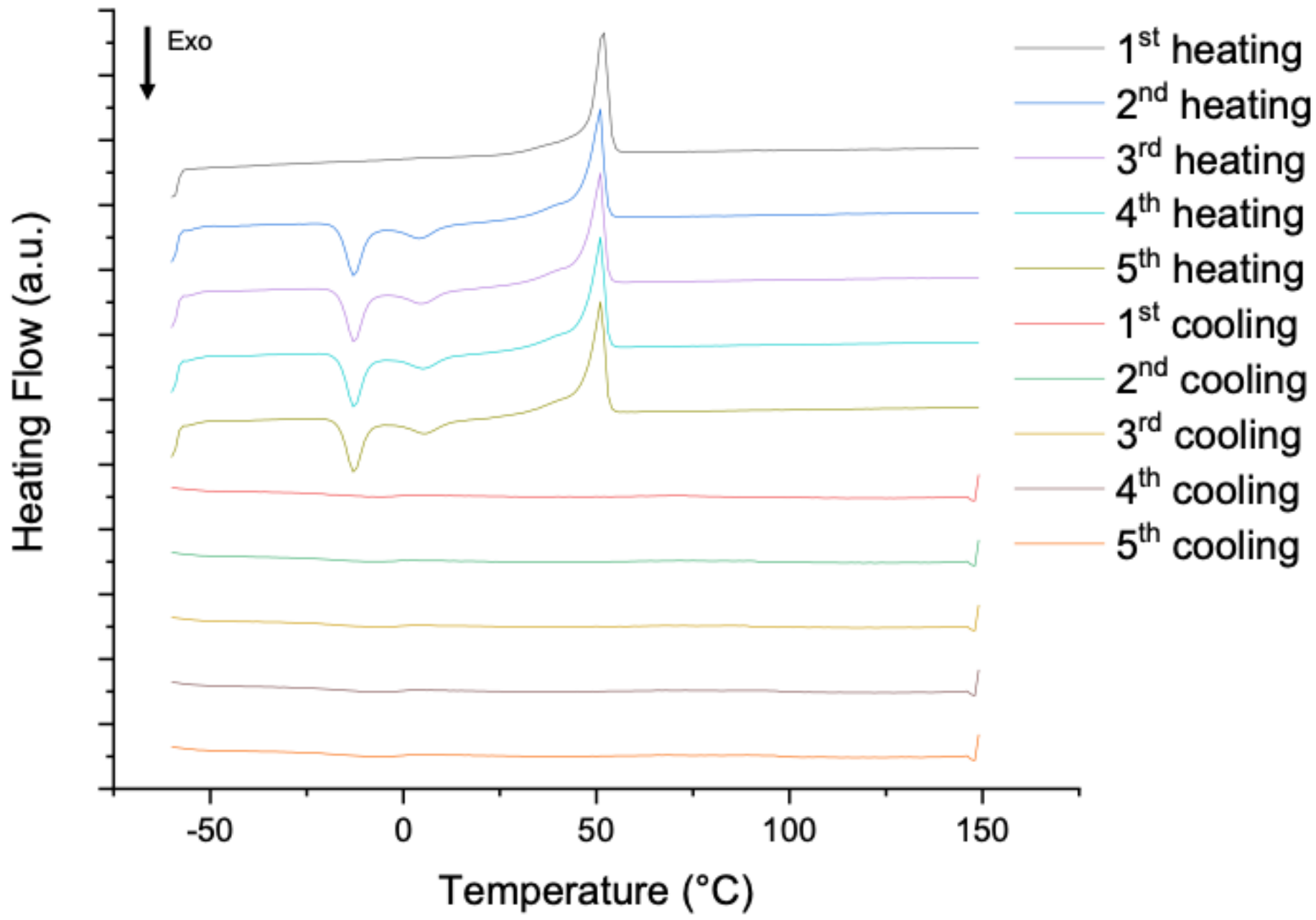

Fig. S13.

Results from Differential Scanning Calorimetry measurements of [P_{66614}][BCB] at a heating rate of 5K/min, measured using a Mettler Toledo DSC3+ STARe, with the sample cold-sealed in air in a sealed aluminium crucible under a nitrogen flow rate of 50 mL/min.

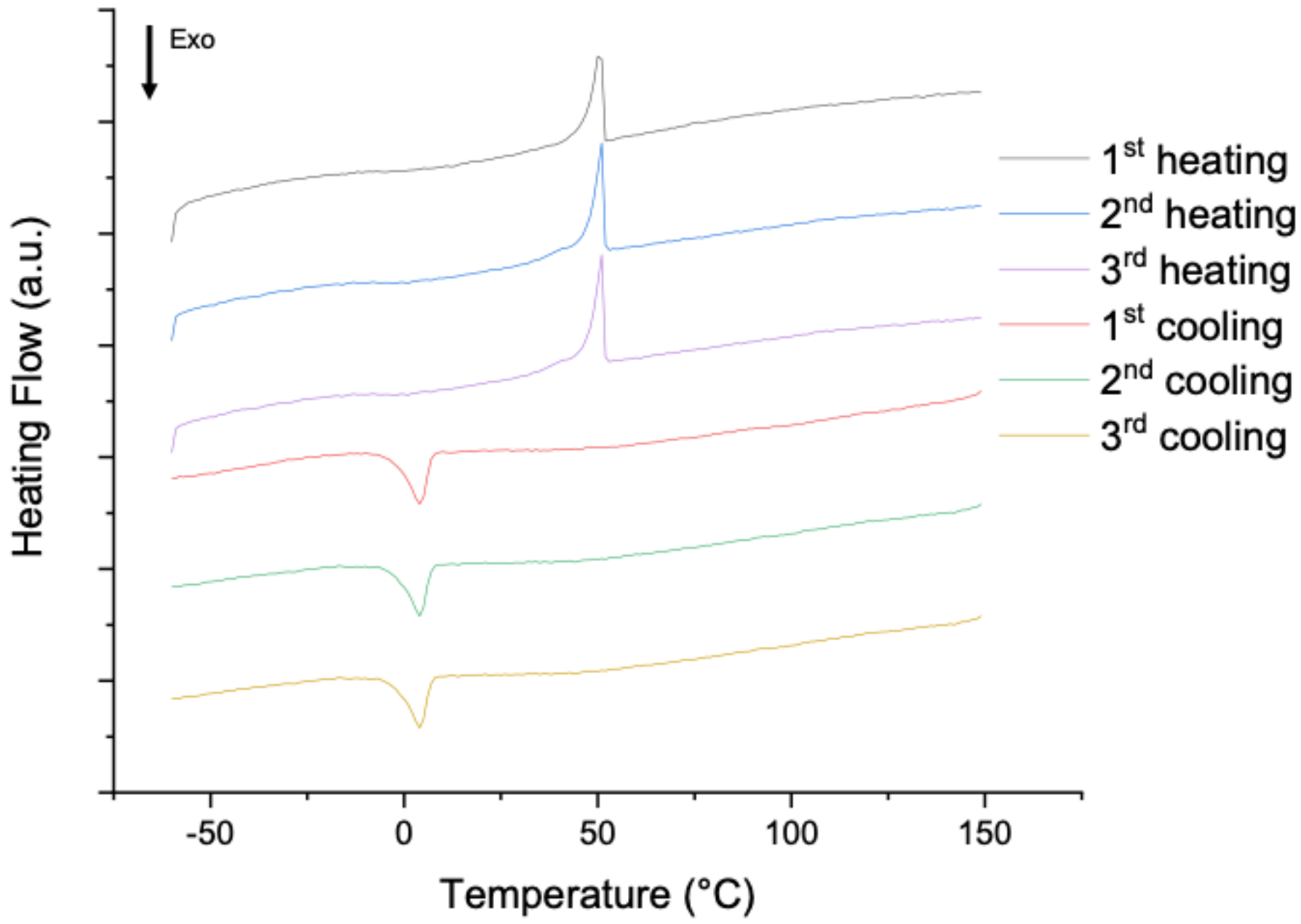

Fig. S14.

Results from Differential Scanning Calorimetry measurements of $[P_{66614}][BCB]$ at a heating rate of 1K/min, measured using a Mettler Toledo DSC3+ STARe, with the sample cold-sealed in air in a sealed aluminium crucible under a nitrogen flow rate of 50 mL/min.

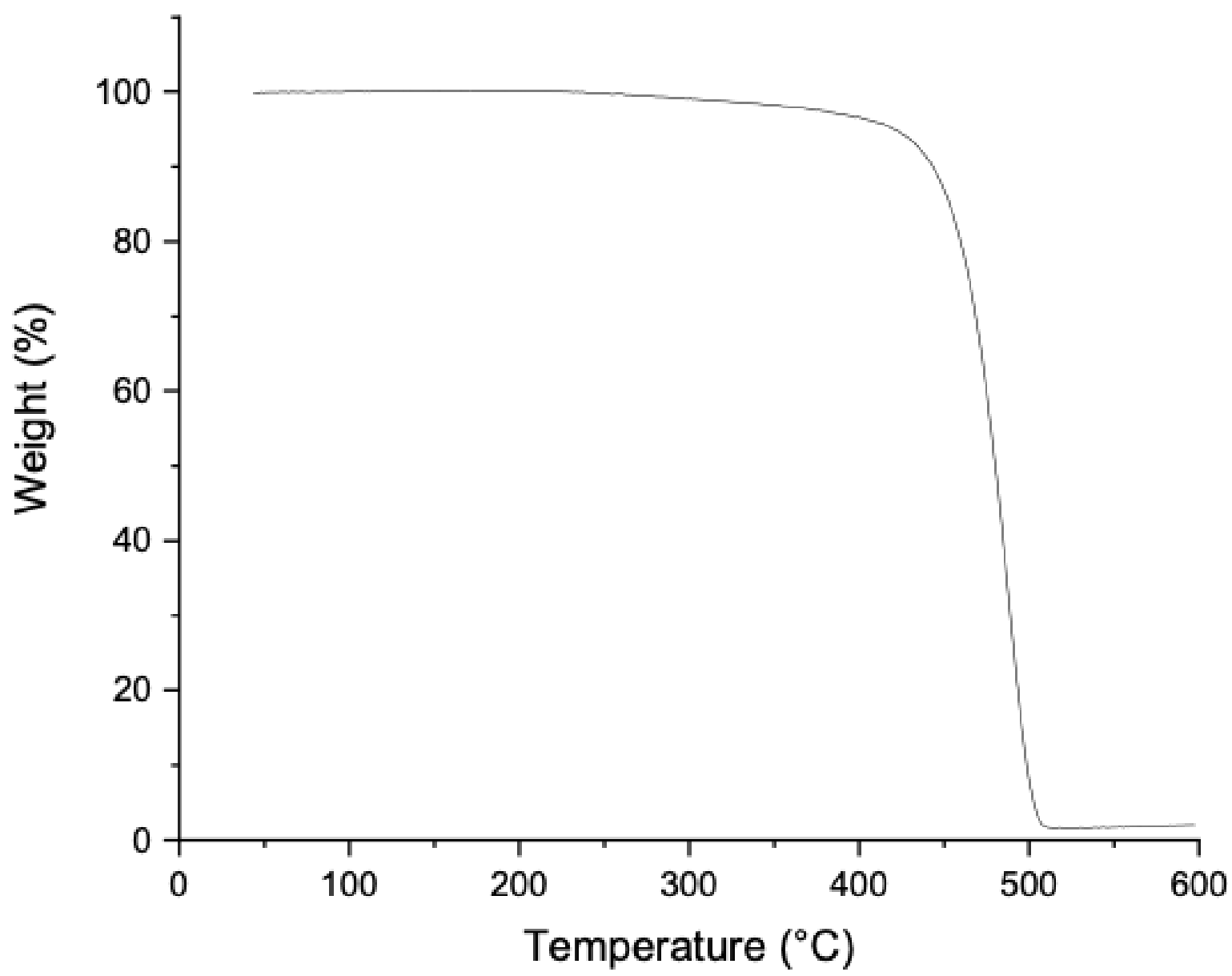

Fig. S15.

Results from thermogravimetric analysis (TGA) of [P_{66614}][BCB], collected using a Netzsch TG 449 F3 Jupiter system with aluminium oxide crucibles, a heating rate of 10 °C/min and nitrogen as purge gas. A 5% onset was used for calculation of degradation temperatures.

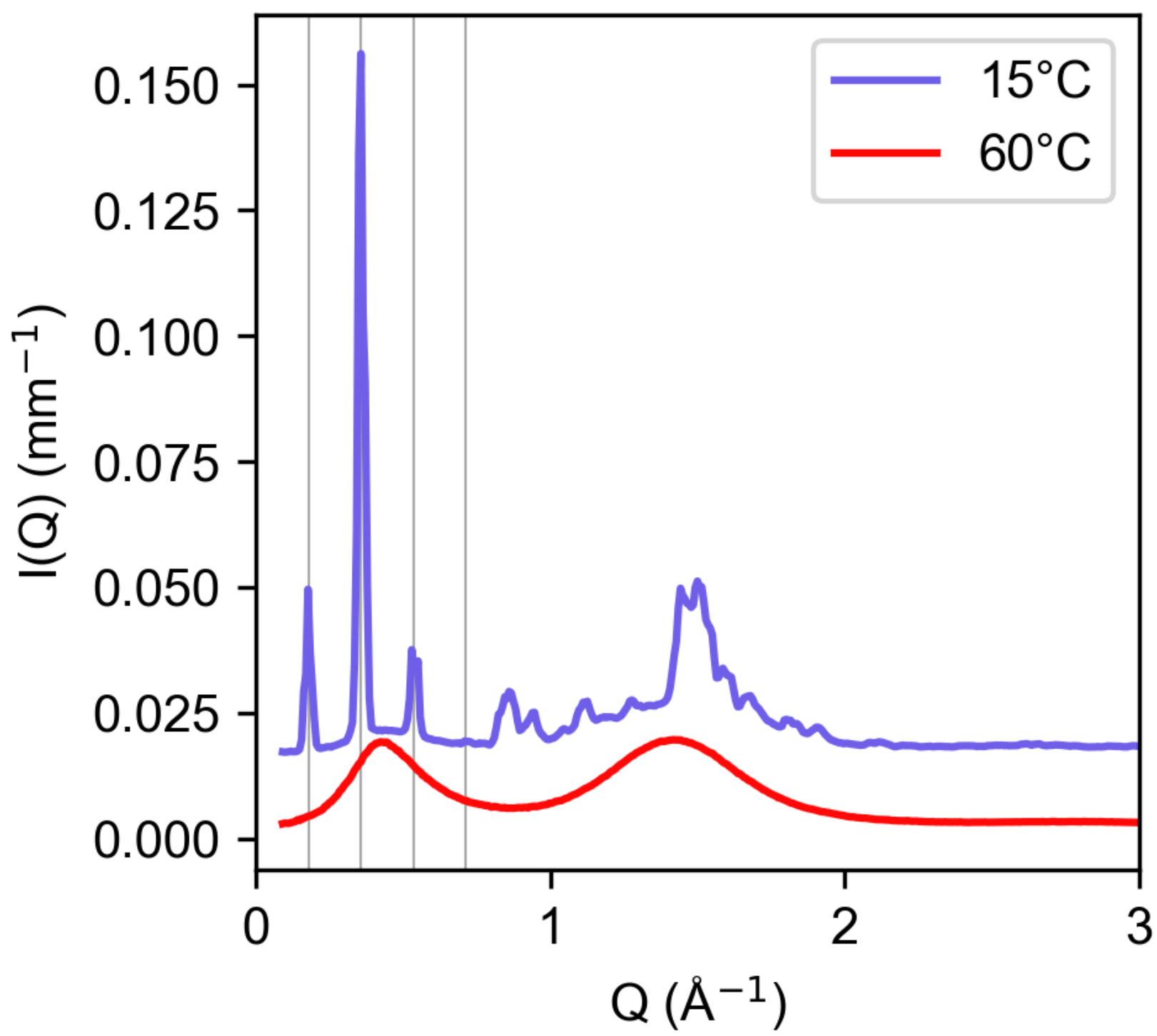

Fig. S16.

SWAXS data for fluid isotropic and sub-ambient semi-solid phases of [P_{66614}][BCB]. The first 4 peaks in the solid sample are indexed to a lamellar-type structure (corresponding to a real space distance of 35.155 ± 0.122 Å), with vertical lines drawn at representative repeating intervals of 0.17794 Å^{-1}.

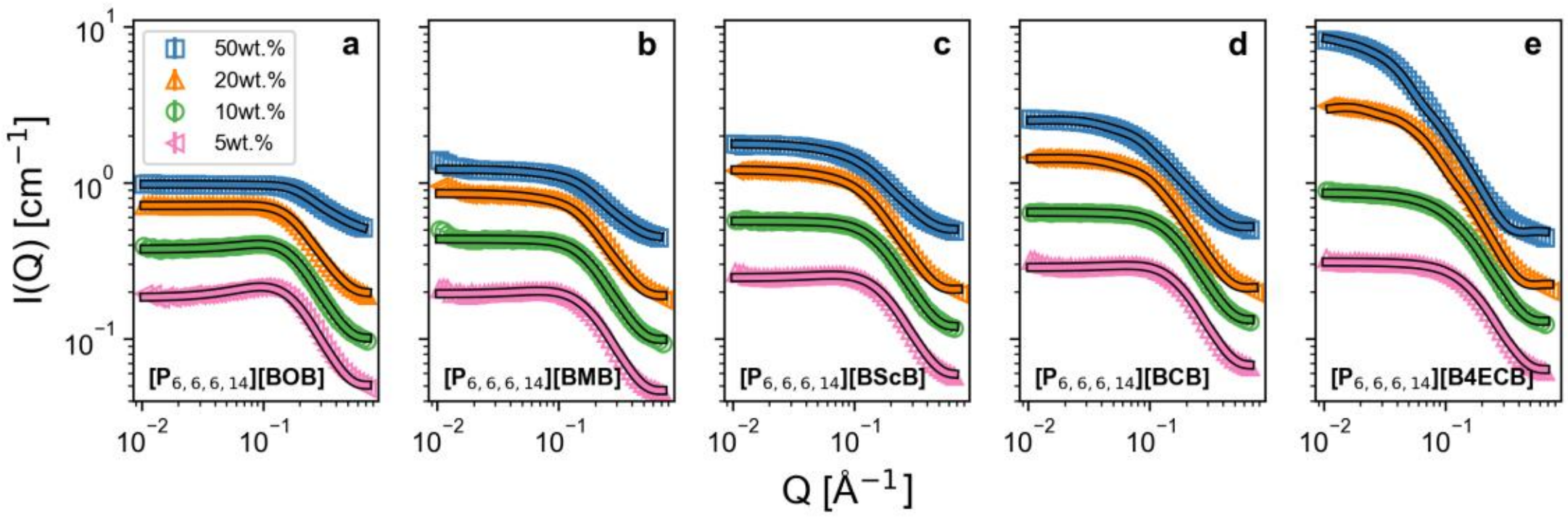

Fig. S17.

(a–e) Small-angle neutron scattering (SANS) data (markers) collected using the BILBY instrument at ANSTO, for the respective ILs dispersed in d_6-propylene carbonate as a function of concentration, and fits (colored lines) to the datasets using the cylinder model with a Hayter-Penfold S(Q).

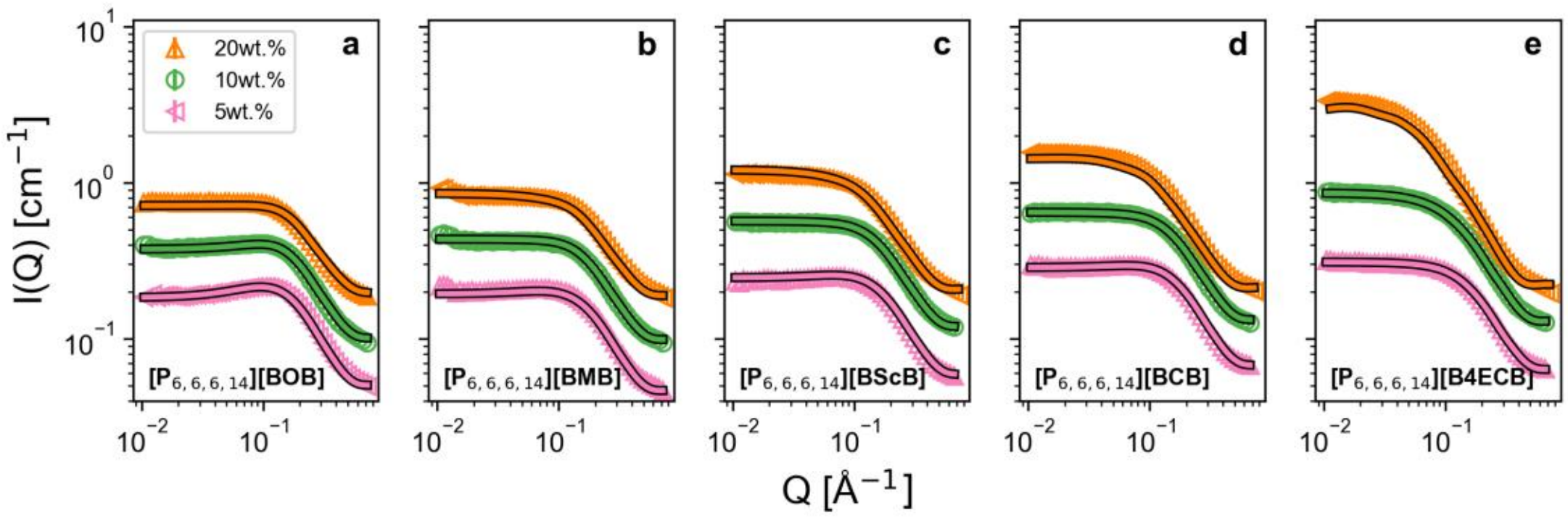

Fig. S18.

(a–e) Small-angle neutron scattering (SANS) data (markers) collected using the BILBY instrument at ANSTO, for the respective ILs dispersed in d_6-propylene carbonate as a function of concentration, reformulated to contain an additional 1 wt.% of D_2O: fits are shown (colored lines) to the data using the cylinder model with a Hayter-Penfold S(Q) (or simple cylinders for B4ECB), fitted to the 'dry' data without correction for hydration, to illustrate (what would be) the minimal effect of any absorbed atmospheric water upon the measured structure.

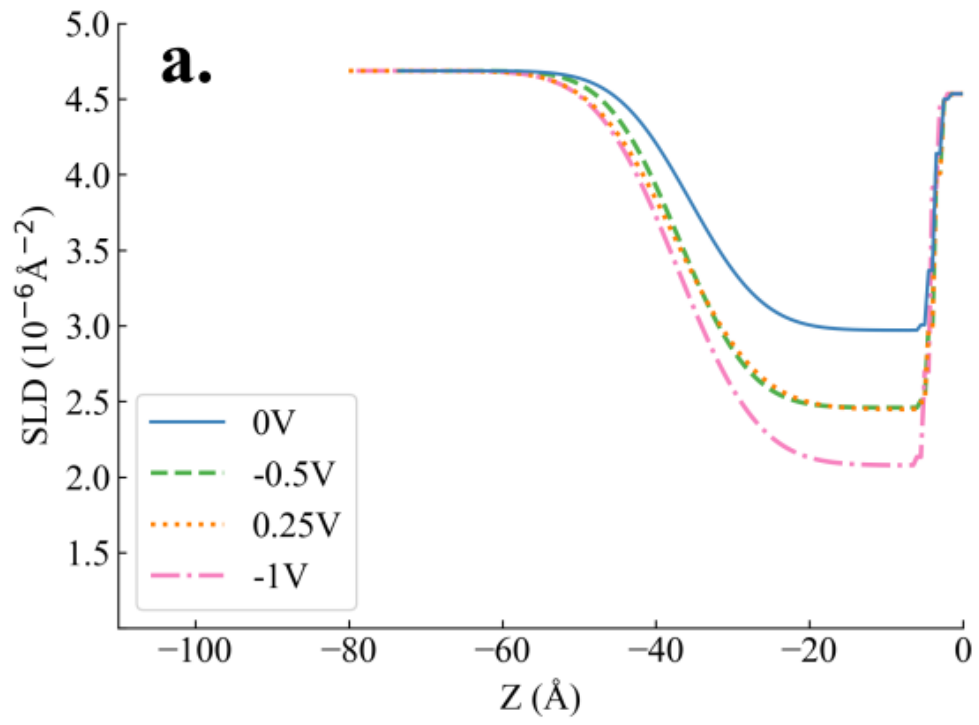

Layer 1				
	0V	-0.5V	0.25V	-1V
Layer Length (Å)	28.8	30.0	30.5	30.2
SLD	2.97	2.46	2.45	2.08
Roughness to bulk	7.6	7.4	8.5	8.4
Roughness to gold	5.0	-	-	-
FOM (e-02)	3.2307	3.0338	3.6573	3.2438

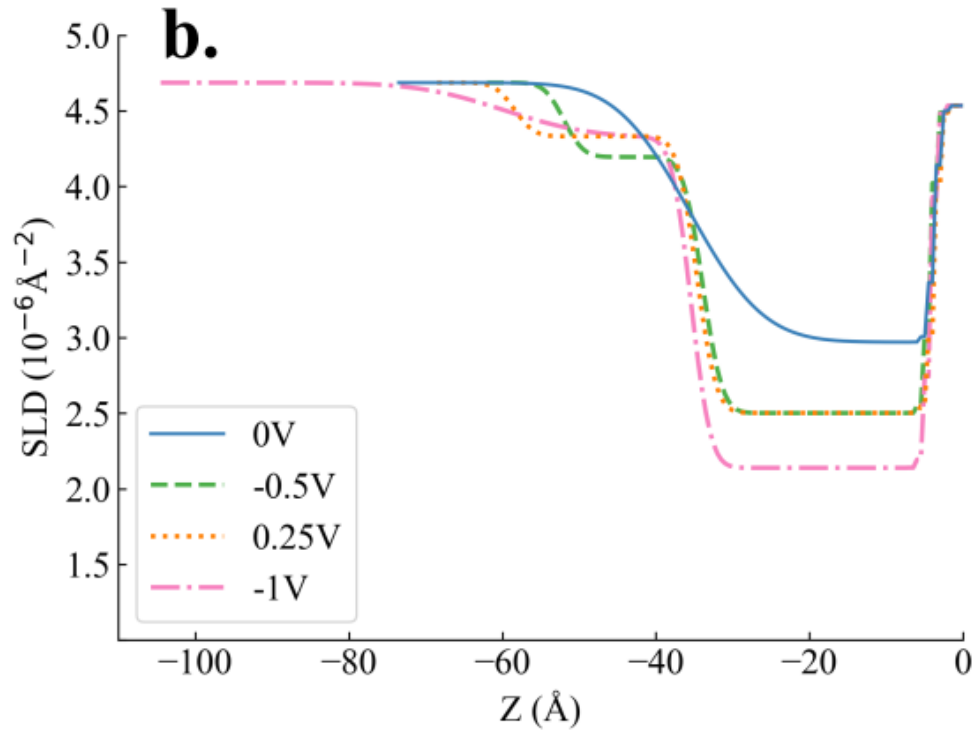

Layer 1				
	0V	-0.5V	0.25V	-1V
Layer Length (Å)	28.8	26.9	27.9	28.3
SLD	2.97	2.50	2.50	2.14
Roughness to bulk/L2	7.6	2.0	2.0	2.0
Roughness to gold	5.0	-	-	-
Layer 2				
Layer Length (Å)		18.1	23.7	24.3
SLD		4.19	4.33	4.32
Roughness to bulk		2.0	2.0	8.9
FOM (e-02)	3.2307	2.9951	3.4875	2.9754

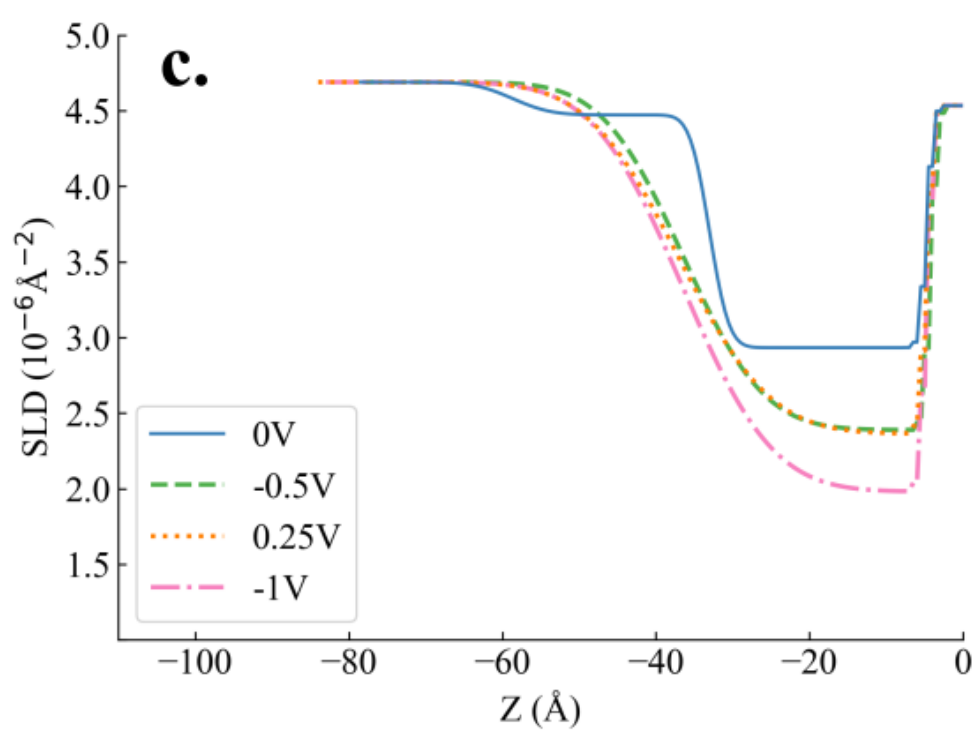

Layer 1				
	0V	-0.5V	0.25V	-1V
Layer Length (Å)	25.4	29.0	29.3	28.9
SLD	2.93	2.39	2.36	1.98
Roughness to bulk/L2	2.0	8.3	9.4	9.3
Roughness to gold	5.0	-	-	-
Layer 2				
Layer Length (Å)	25.7			
SLD	4.47			
Roughness to bulk	4.0			
FOM (e-02)	3.0944	2.9912	3.6095	3.1615

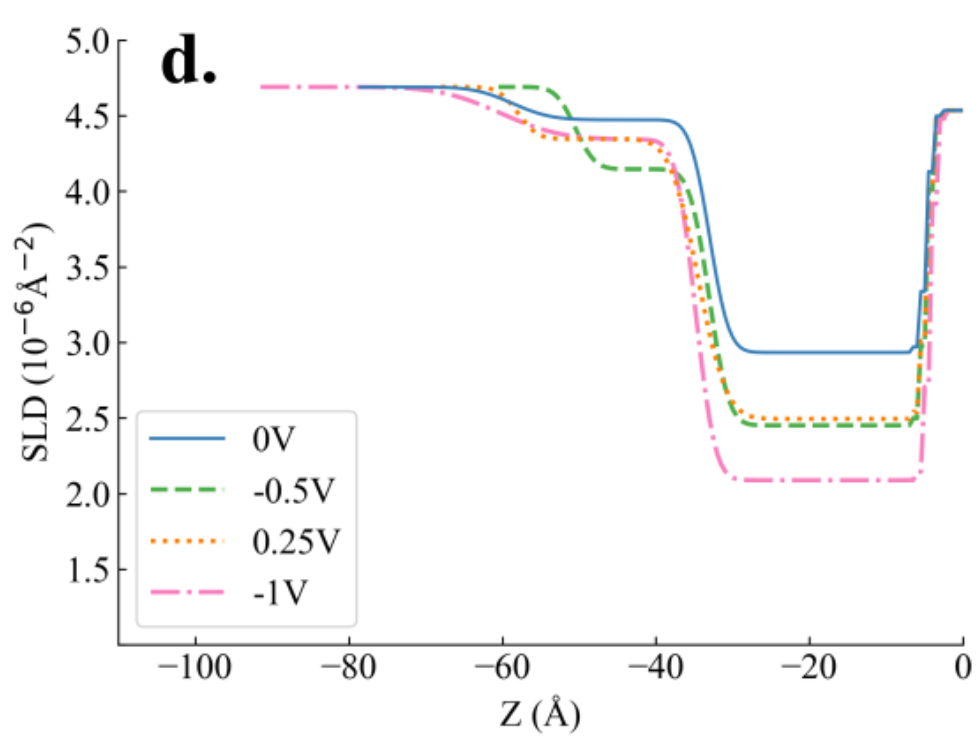

Layer 1				
	0V	-0.5V	0.25V	-1V
Layer Length (Å)	25.4	25.5	26.7	27.4
SLD	2.93	2.45	2.49	2.09
Roughness to L2	2.0	2.0	2.9	2.0
Roughness to gold	5.0	-	-	-
Layer 2				
Layer Length (Å)	25.7	17.4	23.5	25.3
SLD	4.47	4.15	4.34	4.34
Roughness to bulk	4.0	2.0	2.0	6.3
FOM (e-02)	3.0944	2.9678	3.5727	2.9291

Fig. S19.

The SLD plots (left) and their tabulated experimental parameters (right) based on the NR data for [P_{66614}][BCB] (20 wt.% in PC contrast-matched to gold) in the order of 1 (a) and 2 (b) layer models based on a 1 layer 0V fit and 1 (c) and 2 (d) layer models based on a 2 layer 0V fit.

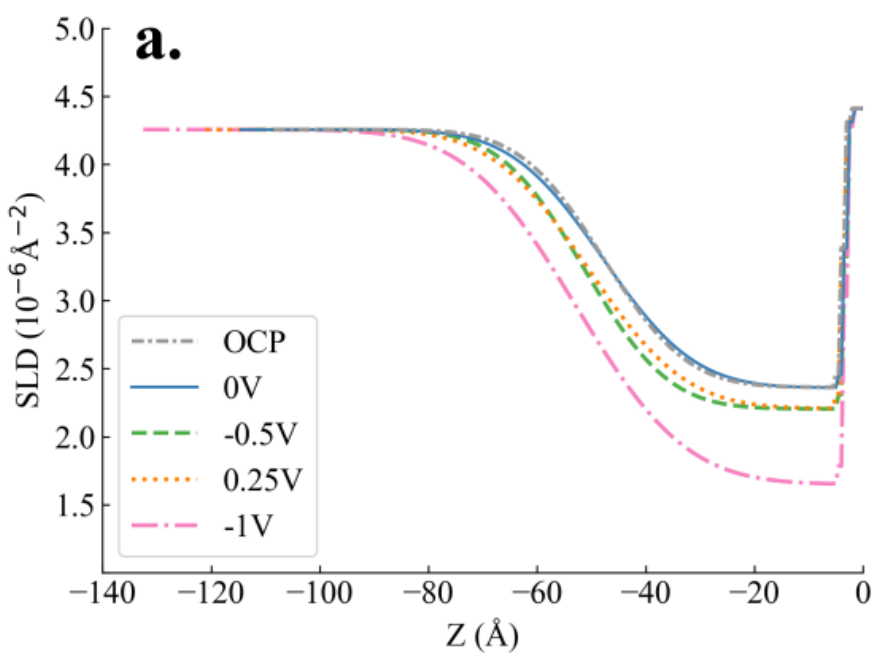

Layer 1					
	OCP	0V	-0.5V	0.25V	-1V
Layer Length (Å)	41.8	41.9	45.4	44.5	47.1
SLD	2.36	2.36	2.20	2.21	1.65
Roughness to bulk	4.3	13.3	12.2	14.1	15.9
Roughness to gold	4	-	-	-	-
FOM (e-02)	3.0267	2.9374	3.1247	2.7748	3.0585

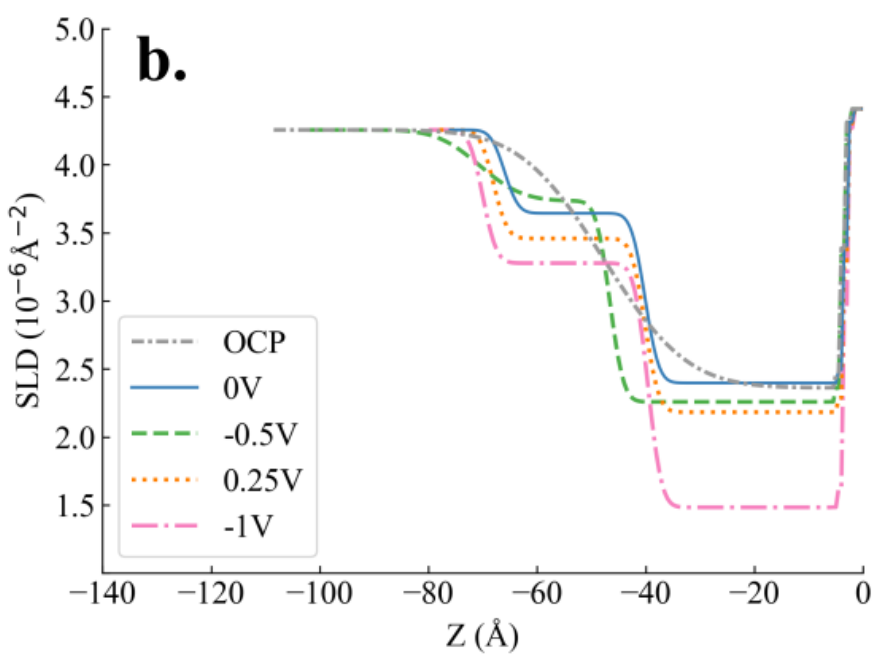

Layer 1					
	OCP	0V	-0.5V	0.25V	-1V
Layer Length (Å)	41.8	34.4	40.6	34.2	33.6
SLD	2.36	2.40	2.26	2.18	1.48
Roughness to L2/bulk	12.1	2.0	2.0	2.0	2.0
Roughness to gold	4.0	-	-	-	-
Layer 2					
Layer Length (Å)		25.9	24.0	27.9	30.4
SLD		3.64	3.73	3.46	3.28
Roughness to bulk		2.0	6.3	2.0	2.0
FOM (e-02)	3.0267	2.9465	3.0803	2.7218	2.4908

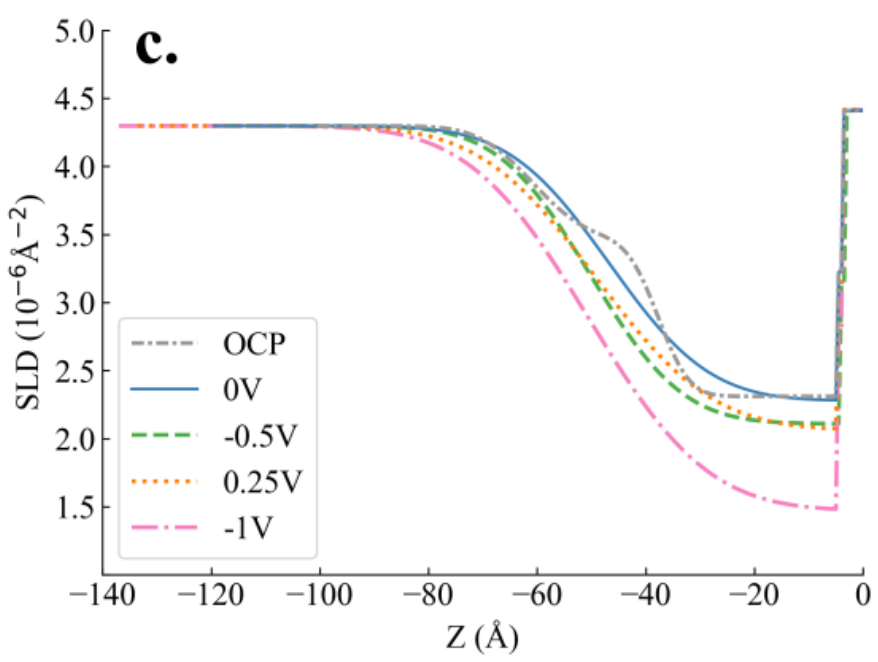

Layer 1					
	OCP	0V	-0.5V	0.25V	-1V
Layer Length (Å)	32.1	41.2	44.7	43.5	44.8
SLD	2.31	2.28	2.11	2.06	1.47
Roughness to bulk/L2	4.5	14.6	13.4	16.9	17.3
Roughness to gold	2.1	-	-	-	-
Layer 2					
Layer Length (Å)	23.1				
SLD	3.46				
Roughness to bulk	7.9				
FOM (e-02)	3.0348	2.8945	2.9277	2.7342	2.7162

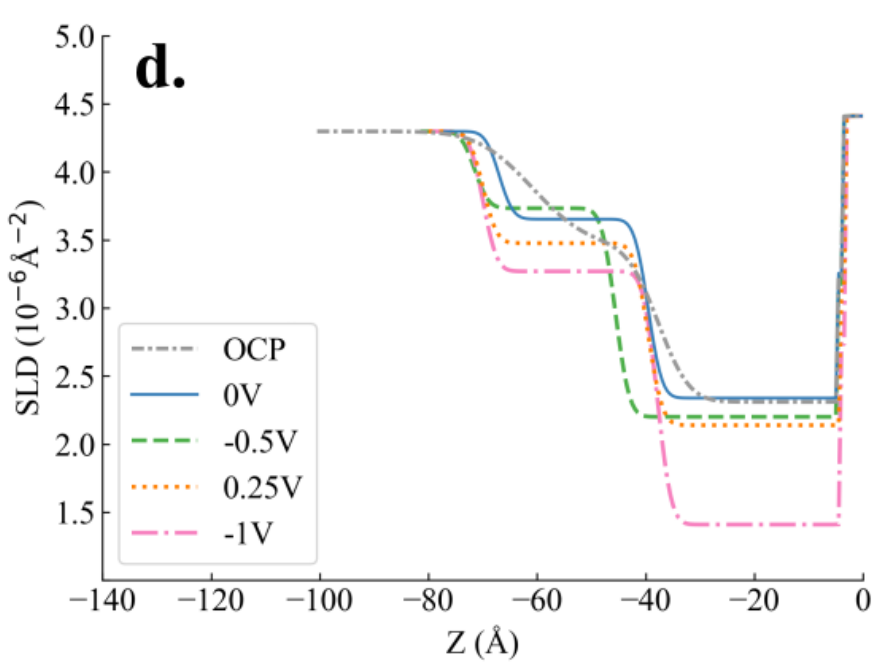

Layer 1					
	OCP	0V	-0.5V	0.25V	-1V
Layer Length (Å)	32.1	33.7	39.8	34.1	32.4
SLD	2.31	2.34	2.20	2.14	1.41
Roughness to L2	4.5	2.0	2.0	2.1	2.0
Roughness to gold	2.1	-	-	-	-
Layer 2					
Layer Length (Å)	23.1	27.4	25.9	30.4	32.1
SLD	3.46	3.65	3.73	3.48	3.27
Roughness to bulk	7.9	2.0	2.0	2.0	2.0
FOM (e-02)	3.0348	2.8737	2.8895	2.6463	2.4270

Fig. S20.

The SLD plots (left) and their tabulated experimental parameters (right) based on the NR data for [P_{66614}][B4ECB] (20 wt.% in PC contrast-matched to gold) in the order of 1 (a) and 2 (b) layer models based on a 1 layer 0V fit and 1 (c) and 2 (d) layer models based on a 2 layer 0V fit.

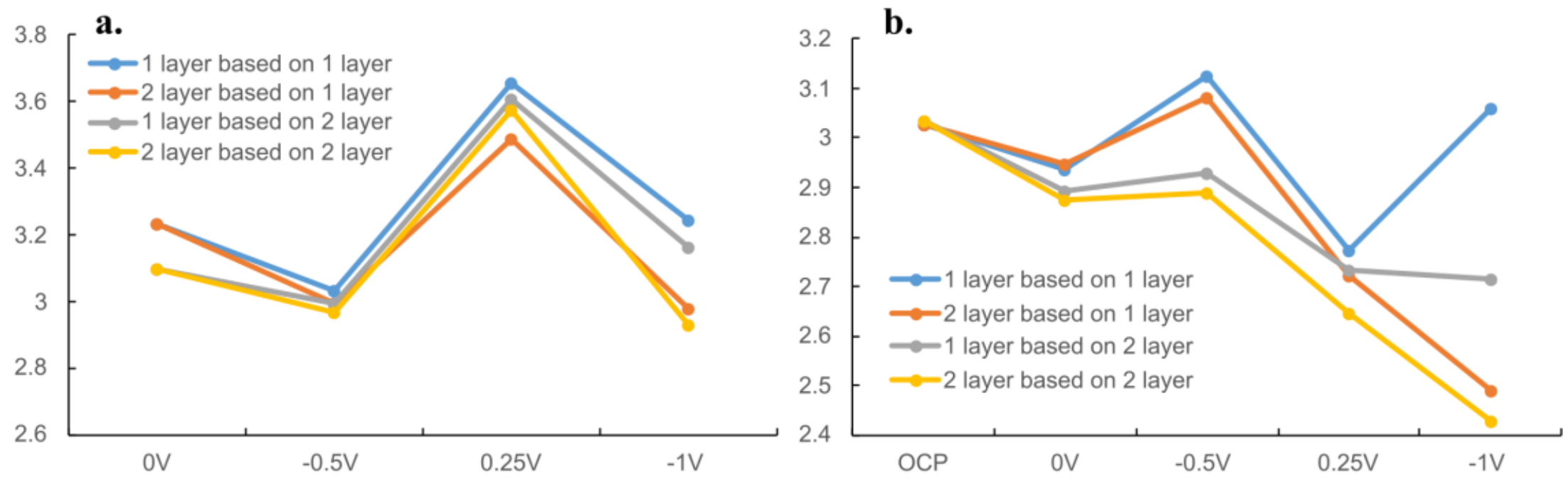

Fig. S21.

The calculated Figure of Merit (FOM, from $\log_{10}[\chi^2]$) representing the quality of the analytical fit on the experimental NR data of (a) [P_{66614}][BCB] and (b) [P_{66614}][B4ECB].

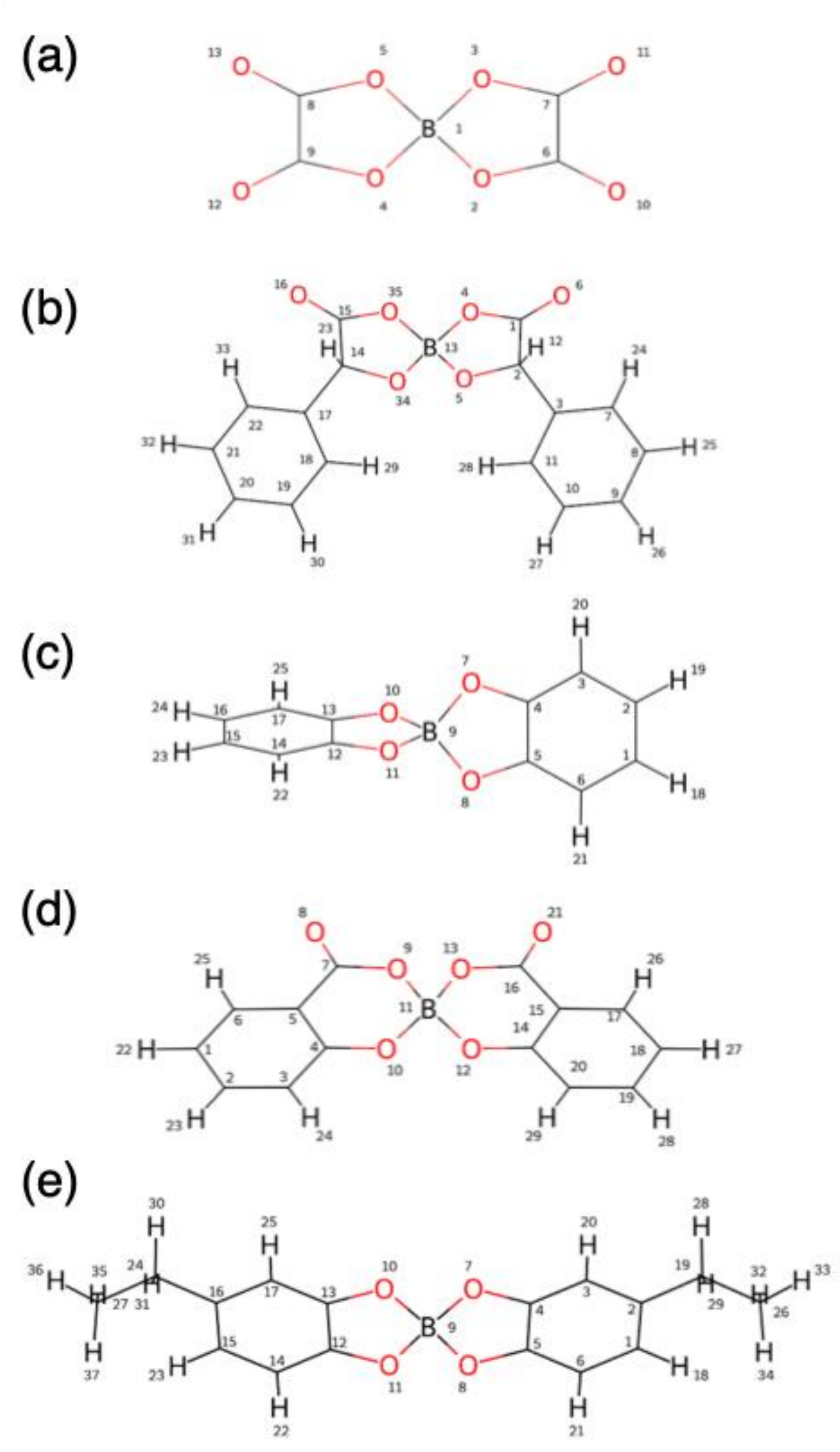

Fig. S22.

Skeletal structures for the five studied orthoborates (a) [BOB], (b) [BMB], (c) [BCB], (d) [BScB] and (e) [B4ECB] with the corresponding atom indices, as used in DFT calculations and corresponding with the tabulated parameters. Structures were directly generated from .xyz files using the *rdkit* Python module.

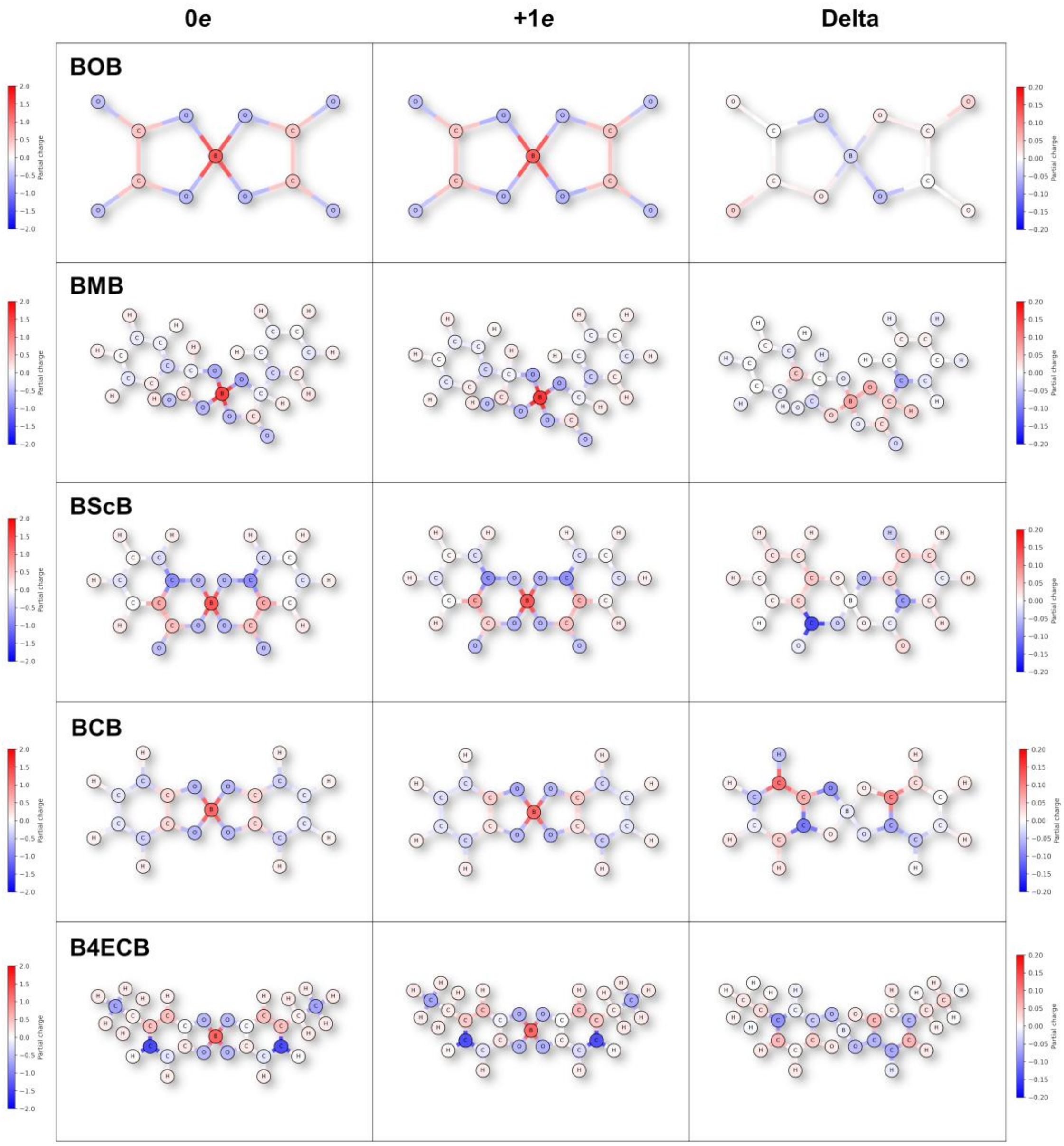

Fig. S23.

Cartoons showing calculated DFT charges for the five studied orthoborates (a) BOB, (b) BMB, (c) BCB, (d) BScB and (e) B4ECB, as shown in Figure S22 and calculated at the M06-2X/6-31++G** level. The base Mulliken charge (q) is provided in the first column, alongside the calculated Mulliken charge when the anion was subjected to a +1 point charge (q_{+1}) at a distance of 5 Å from the B centre in column 2; the difference in charge (Δq) is then shown in column 3. Charges were colormapped using bounds of +/-2e (for 0e and 1e samples) and /-0.2e for the difference plots in the third column; the colormapping is provided alongside. Structures were drawn from SMILES strings using the rdkit Python module.

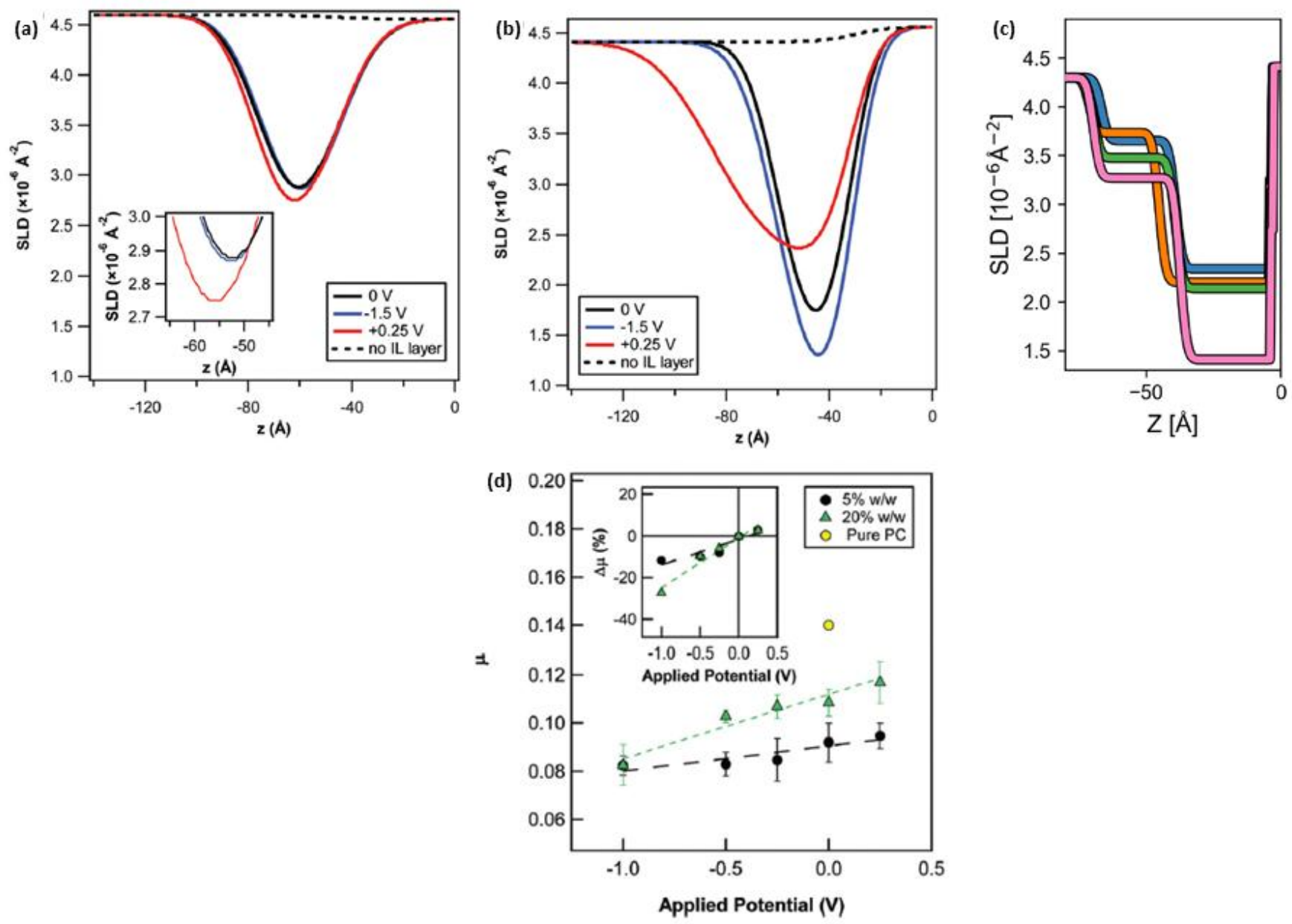

Fig. S24.

A comparison of the electroresponsivity of ILs by their measured SLD; (a) 5 wt% [P_{66614}][BMB], (b) 20 wt% [P_{66614}][BMB] and (c) 20 wt% [P_{66614}][B4ECB]. The connection between electroresponsivity and friction coefficient is made evident in (d) showing significantly reduced friction at 20 wt% [P_{66614}][BMB] as compared to 5 wt% [P_{66614}][BMB]. Figures (a), (b) and (d) are reproduced from Pilkington *et al*. *(21)* under a Creative Commons CC BY-NC 3.0 license (https://creativecommons.org/licenses/by-nc/3.0/).

Table S1.

Peak integrals of SWAXS data, where D_1 = 0.00 – 0.86 Å^{-1}, D_2 = 0.86 – 2.50 Å^{-1}, and D_3 = 2.50 – 3.32 Å^{-1}.

	Peak Integral			
Anion	D_1	D_2	D_3	$D_1/\Sigma D_i$
[BOB]	0.00736	0.01822	0.00309	0.257
[BMB]	0.00745	0.01537	0.00249	0.294
[BScB]	0.00801	0.01512	0.00296	0.307
[BCB]	0.00794	0.01456	0.00266	0.316
[B4ECB]	0.00977	0.01599	0.00312	0.338

Table S2.

Calculated SANS fit parameters, for fits using the cylinder model with a Hayter-Penfold S(Q), showing the incoherent background c_0, “aggregate” radius (*R*) and Length (*L*), effective radius (R_{eff}) used for *S(Q)* calculation (the structure factor), the volume fraction of the Hayter-Penfold structure factor ($\Phi_{S(Q)}$), and the goodness of fit parameter (χ^2). For reference, the calculated accurate SANS sample compositions are also provided as weight percentage, volume fraction, and mole fraction of IL.

Anion	Wt.%	[IL] in PC Φ_{IL}	X_{IL}	c_0 (cm^{-1})	R (Å)	L (Å)	R_{eff} (Å)	$\Phi_{S(Q)}$	χ^2
[BOB]	5	0.062	0.008	0.050	5.59±0.01	46.20±0.50	12.6	0.093	13.6
	9.9	0.122	0.017	0.101	5.45±0.01	48.30±0.43	12.7	0.091	33.2
	20.1	0.241	0.039	0.197	5.24±0.01	52.63±0.34	13.1	0.089	103.9
	49.5	0.553	0.136	0.494	3.84±0.02	42.05±0.97	10.0	0.059	4.2
[BMB]	5	0.061	0.007	0.046	5.76±0.02	46.42±0.59	12.8	0.083	11.3
	10	0.120	0.015	0.099	5.68±0.01	51.33±0.54	13.4	0.076	54.4
	19.9	0.234	0.033	0.189	5.64±0.01	51.16±0.62	13.3	0.046	148.6
	44.2	0.493	0.097	0.449	5.14±0.01	48.66±0.49	12.4	0.045	44.9
[BScB]	5	0.061	0.007	0.059	5.74±0.01	48.36±0.50	13.1	0.082	10.9
	10	0.122	0.015	0.120	5.82±0.01	55.31±0.47	14.1	0.072	41
	20	0.238	0.034	0.207	6.00±0.01	62.08±0.41	15.2	0.035	288
	45.9	0.514	0.107	0.502	5.72±0.01	62.37±0.48	14.9	0.036	133.9
[BCB]	5	0.062	0.008	0.068	5.86±0.01	47.10±0.47	13.0	0.080	13.8
	10	0.122	0.017	0.132	5.92±0.01	56.20±0.48	14.3	0.069	46.4
	19.9	0.237	0.036	0.211	6.14±0.00	102.98±0.47	20.3	0.085	408.2
	45.1	0.505	0.111	0.521	6.23±0.01	125.21±0.76	22.9	0.077	359.1
[B4ECB]	5	0.058	0.007	0.063	6.05±0.01	45.65±0.61	13.0	0.046	14.3
	10	0.115	0.015	0.129	6.31±0.01	55.37±0.69	14.7	0.026	102.6
	20	0.226	0.034	0.221	7.26±0.00	265.76±1.33	38.3	0.063	1531.4
	43.8	0.476	0.099	0.482	8.93±0.00	573.86±1.89	67.1	0.073	5249

Table S3.

Density, molecular formula, SLD, molar mass and molecular volumes of the bulk IL and its constituent ions, and PC.

Component	Formula	ρ (g/mL)	SLD (×10^6 Å^{-2})	M (g/mol)	V_m (Å^3)
$[P_{66614}]^+$	$PC_{32}H_{68}$	0.880	−0.400	483.85	914
$[BCB]^-$	$BO_4C_{12}H_8$	1.602	3.331	227.00	235
$[P_{66614}][BCB]$	$PBC_{44}H_{76}O_4$	1.028	0.365	710.85	1148
$[B4ECB]^-$	$BO_4C_{16}H_{16}$	1.305	2.083	283.11	360
$[P_{66614}][B4ECB]$	$PBC_{48}H_{84}O_4$	1.081	0.327	766.96	1178
d-PC	$C_4D_6O_3$	1.258	5.869	108.13	142

Table S4.

Results from DFT calculations of the bis(oxalato)borate [BOB] anion as shown in Fig. S22a. For Tables S4-S8: these values were optimized using DFT at the M06-2X/6-31++G** level. The base Mulliken charge (q) is provided, alongside the calculated Mulliken charge when the anion was subjected to a +1 point charge (q_{+1}) at a distance of 5Å from the B centre; for reference, the difference in charge (Δq) is also shown.

Atom	Type	q (e)	q_{+1} (e)	Δq
1	B	1.311	1.279	-0.032
2	O	-0.543	-0.590	-0.047
3	O	-0.543	-0.529	0.014
4	O	-0.543	-0.529	0.014
5	O	-0.543	-0.591	-0.048
6	C	0.445	0.444	-0.001
7	C	0.445	0.454	0.010
8	C	0.445	0.444	0.000
9	C	0.445	0.454	0.009
10	O	-0.479	-0.471	0.009
11	O	-0.479	-0.448	0.032
12	O	-0.479	-0.448	0.032
13	O	-0.479	-0.471	0.009

Table S5.

Results from DFT calculations of the bis(mandelato)borate [BMB] anion (Fig. S22b).

Atom	Type	q (e)	q_{+1} (e)	Δq
1	C	0.207	0.233	-0.026
2	C	-0.123	-0.087	-0.035
3	C	-0.314	-0.383	0.069
4	O	-0.517	-0.533	0.015
5	O	-0.718	-0.657	-0.060
6	O	-0.440	-0.468	0.027
7	C	0.150	0.132	0.018
8	C	-0.182	-0.183	0.001
9	C	0.028	0.039	-0.011
10	C	-0.108	-0.099	-0.010
11	C	-0.078	-0.082	0.005
12	H	0.131	0.168	-0.037
13	B	1.497	1.566	-0.069
14	C	-0.095	-0.093	-0.002
15	C	0.251	0.223	0.028
16	O	-0.454	-0.467	0.013
17	C	-0.338	-0.298	-0.040
18	C	-0.100	-0.116	0.016
19	C	-0.150	-0.144	-0.006
20	C	0.037	0.033	0.004
21	C	-0.179	-0.176	-0.003
22	C	0.154	0.141	0.013
23	H	0.184	0.167	0.016
24	H	0.190	0.185	0.006
25	H	0.133	0.117	0.016
26	H	0.132	0.116	0.016
27	H	0.151	0.141	0.011
28	H	0.079	0.080	-0.001
29	H	0.074	0.072	0.002
30	H	0.143	0.143	0.000
31	H	0.129	0.116	0.013
32	H	0.133	0.118	0.015
33	H	0.188	0.178	0.010
34	O	-0.636	-0.651	0.015
35	O	-0.559	-0.531	-0.027

Table S6.

Results from DFT calculations of the bis(salicylato)borate [BScB] anion (Fig. S22d).

Atom	Type	q (e)	q_{+1} (e)	Δq
1	C	-0.214	-0.205	-0.008
2	C	0.003	0.013	-0.010
3	C	-0.266	-0.245	-0.021
4	C	-0.888	-0.845	-0.043
5	C	0.660	0.691	-0.031
6	C	0.057	0.077	-0.021
7	C	0.490	0.346	0.145
8	O	-0.486	-0.500	0.014
9	O	-0.543	-0.577	0.035
10	O	-0.534	-0.522	-0.012
11	B	1.324	1.323	0.001
12	O	-0.534	-0.588	0.055
13	O	-0.543	-0.533	-0.010
14	C	-0.888	-0.854	-0.033
15	C	0.660	0.582	0.078
16	C	0.490	0.478	0.013
17	C	0.057	0.074	-0.017
18	C	-0.214	-0.228	0.014
19	C	0.003	0.032	-0.029
20	C	-0.266	-0.228	-0.038
21	O	-0.486	-0.458	-0.027
22	H	0.114	0.129	-0.015
23	H	0.126	0.144	-0.018
24	H	0.139	0.152	-0.013
25	H	0.178	0.177	0.001
26	H	0.178	0.194	-0.016
27	H	0.114	0.133	-0.019
28	H	0.126	0.137	-0.011
29	H	0.139	0.104	0.036

Table S7.
Results from DFT calculations of the bis(catecholato)borate [BCB] anion (Fig. S22c).

Atom	Type	q (e)	q_{+1} (e)	Δq
1	C	-0.184	-0.197	-0.013
2	C	-0.184	-0.185	-0.002
3	C	-0.349	-0.323	0.026
4	C	0.297	0.392	0.095
5	C	0.297	0.226	-0.072
6	C	-0.349	-0.391	-0.042
7	O	-0.558	-0.546	0.011
8	O	-0.558	-0.578	-0.020
9	B	1.201	1.187	-0.015
10	O	-0.558	-0.644	-0.087
11	O	-0.558	-0.542	0.015
12	C	0.297	0.194	-0.103
13	C	0.297	0.361	0.063
14	C	-0.349	-0.312	0.037
15	C	-0.184	-0.197	-0.013
16	C	-0.184	-0.234	-0.050
17	C	-0.349	-0.235	0.114
18	H	0.106	0.121	0.015
19	H	0.106	0.124	0.018
20	H	0.136	0.151	0.015
21	H	0.136	0.141	0.005
22	H	0.136	0.156	0.020
23	H	0.106	0.126	0.020
24	H	0.106	0.118	0.012
25	H	0.136	0.089	-0.047

Table S8.

Results from DFT calculations of the bis(4-ethylcatecholato)borate [B4ECB] anion (Fig. S22e).

Atom	Type	q (e)	q_{+1} (e)	Δq
1	C	-1.419	-1.364	-0.055
2	C	0.487	0.430	0.057
3	C	0.498	0.506	-0.008
4	C	-0.047	0.006	-0.054
5	C	0.158	0.101	0.058
6	C	-0.202	-0.268	0.066
7	O	-0.545	-0.527	-0.018
8	O	-0.549	-0.597	0.049
9	B	1.214	1.202	0.011
10	O	-0.545	-0.598	0.053
11	O	-0.549	-0.534	-0.015
12	C	0.158	0.194	-0.036
13	C	-0.047	-0.087	0.039
14	C	-0.202	-0.195	-0.008
15	C	-1.419	-1.366	-0.054
16	C	0.486	0.402	0.084
17	C	0.498	0.488	0.009
18	H	0.046	0.062	-0.016
19	C	0.109	0.130	-0.021
20	H	0.127	0.145	-0.018
21	H	0.147	0.131	0.015
22	H	0.147	0.163	-0.016
23	H	0.046	0.064	-0.018
24	C	0.109	0.135	-0.026
25	H	0.127	0.116	0.012
26	C	-0.710	-0.679	-0.032
27	C	-0.710	-0.692	-0.019
28	H	0.158	0.164	-0.006
29	H	0.159	0.169	-0.011
30	H	0.158	0.164	-0.006
31	H	0.159	0.154	0.005
32	H	0.166	0.159	0.007
33	H	0.145	0.158	-0.013
34	H	0.166	0.169	-0.003
35	H	0.166	0.173	-0.007
36	H	0.145	0.154	-0.009
37	H	0.166	0.166	0.000

Table S9.

Results from polarization sub-routines performed upon the DFT-optimized anion structures at the M06-2X/6-31++G** level, determined from the principal component polarizability tensors. Both isotropic polarizability $<\alpha>$ and the anisotropic polarizability $\Delta\alpha$ are shown; the anisotropic polarizability volume was divided by the calculated ion surface area to estimate the size-normalized polarizability trend, $\Delta\alpha/A$. Finally, the root mean square for the per-anion Δq datasets in Tables S4-8 is provided ($RMS_{\Delta q}$), effectively a proxy for the response to polarization effects via the mean magnitude of charge shifts.

Anion	$<\alpha>$ (Å^3)	$\Delta\alpha$ (Å^3)	A (Å^2)	$\Delta\alpha/A$ (Å)	$RMS_{\Delta q}$
[BOB]	11.952	8.266	307	0.0269	0.091
[BMB]	31.252	18.125	598	0.0303	0.153
[BScB]	29.018	16.271	510	0.0319	0.207
[BCB]	25.384	17.755	429	0.0414	0.247
[B4ECB]	33.312	24.264	583	0.0416	0.200

REFERENCES

1. R. D. Rogers, K. R. Seddon, Ionic liquids—Solvents of the future? *Science* **302**, 792–793 (2003).

2. P. G. Jessop, Searching for green solvents. *Green Chem.* **13**, 1391–1398 (2011).

3. C. J. Clarke, W. C. Tu, O. Levers, A. Bröhl, J. P. Hallett, Green and sustainable solvents in chemical processes. *Chem. Rev.* **118**, 747–800 (2018).

4. A. J. Greer, J. Jacquemin, C. Hardacre, Industrial applications of ionic liquids. *Molecules* **25**, 5207 (2020).

5. A. E. Somers, B. Khemchandani, P. C. Howlett, J. Sun, D. R. MacFarlane, M. Forsyth, Ionic liquids as antiwear additives in base oils: Influence of structure on miscibility and antiwear performance for steel on aluminum. *ACS Appl. Mater. Interfaces* **5**, 11544–11553 (2013).

6. C. Ye, W. Liu, Y. Chen, L. Yu, Room-temperature ionic liquids: A novel versatile lubricant. *Chem. Commun.* **2001**, 2244–2245 (2001).

7. N. Hjalmarsson, E. Bergendal, Y.-L. Wang, B. Munavirov, D. Wallinder, S. Glavatskih, T. Aastrup, R. Atkin, I. Furó, M. W. Rutland, Electro-responsive surface composition and kinetics of an ionic liquid in a polar oil. *Langmuir* **35**, 15692–15700 (2019).

8. P. Rohlmann, S. Watanabe, M. R. Shimpi, J. Leckner, M. W. Rutland, J. B. Harper, S. Glavatskih, Boundary lubricity of phosphonium bisoxalatoborate ionic liquids. *Tribol. Int.* **161**, 107075 (2021).

9. A. B. Reddy, G. A. Pilkington, M. W. Rutland, S. Glavatskih, Tribotronic control of an ionic boundary layer in operando extends the limits of lubrication. *Sci. Rep.* **12**, 20479 (2022).

10. B. Munavirov, J. J. Black, F. U. Shah, J. Leckner, M. W. Rutland, J. B. Harper, S. Glavatskih, The effect of anion architecture on the lubrication chemistry of phosphonium orthoborate ionic liquids. *Sci. Rep.* **11**, 24021 (2021).

11. P. Rohlmann, J. J. Black, S. Watanabe, J. Leckner, M. R. Shimpi, M. W. Rutland, J. B. Harper, S. Glavatskih, Tribochemistry of imidazolium and phosphonium bis(oxalato)borate ionic liquids: Understanding the differences. *Tribol. Int.* **181**, 108263 (2023).

12. P. Rohlmann, B. Munavirov, I. Furó, O. N. Antzutkin, M. W. Rutland, S. Glavatskih, Non-halogenated ionic liquid dramatically enhances tribological performance of biodegradable oils. *Front. Chem.* **7**, 98 (2019).

13. X. Mao, P. Brown, C. Červinka, G. Hazell, H. Li, Y. Ren, D. Chen, R. Atkin, J. Eastoe, I. Grillo, A. A. H. Padua, M. F. Costa Gomes, T. A. Hatton, Self-assembled nanostructures in ionic liquids facilitate charge storage at electrified interfaces. *Nat. Mater.* **18**, 1350–1357 (2019).

14. A. Elbourne, M. N. Awad, L. Meredith, A. J. Christofferson, T. L. Greaves, G. Bryant, S. J. Bryant, "Solid-liquid interfacial nanostructure of ionic liquids and deep eutectic solvents," in *Encyclopedia of Solid-Liquid Interfaces* (Elsevier, ed. 1, 2024), pp. 627–650.

15. A. B. Reddy, B. Munavirov, G. A. Pilkington, G. Calderon Salmeron, M. W. Rutland, S. Glavatskih, Micro- to nano- and from surface to bulk: Influence of halogen-free ionic liquid architecture and dissociation on green oil lubricity. *ACS Sustain Chem. Eng.* **9**, 13606–13617 (2021).

16. F. U. Shah, S. Glavatskih, O. N. Antzutkin, Boron in tribology: From borates to ionic liquids. *Tribol. Lett.* **51**, 281–301 (2013).

17. F. U. Shah, A. Holmgren, M. W. Rutland, S. Glavatskih, O. N. Antzutkin, Interfacial behavior of orthoborate ionic liquids at inorganic oxide surfaces probed by NMR, IR, and Raman spectroscopy. *J. Phys. Chem. C* **122**, 18523–18535 (2018).

18. S. Watanabe, G. A. Pilkington, A. Oleshkevych, P. Pedraz, M. Radiom, R. Welbourn, S. Glavatskih, M. W. Rutland, Interfacial structuring of non-halogenated imidazolium ionic liquids at charged surfaces: Effect of alkyl chain length. *Phys. Chem. Chem. Phys.* **22**, 8450–8460 (2020).

19. O. S. Hammond, G. Bousrez, F. Mehler, S. Li, M. R. Shimpi, J. Doutch, L. Cavalcanti, S. Glavatskih, O. N. Antzutkin, M. W. Rutland, A.-V. Mudring, Molecular architecture effects on bulk nanostructure in bis(orthoborate) ionic liquids. *Small* **19**, e2300912 (2023).

20. S. Li, O. S. Hammond, A. Nelson, L. de Campo, M. Moir, C. Recsei, M. R. Shimpi, S. Glavatskih, G. A. Pilkington, A.-V. Mudring, M. W. Rutland, Anion architecture controls structure and electroresponsivity of anhalogenous ionic liquids in a sustainable fluid. *J. Phys. Chem. B* **128**, 4231–4242 (2024).

21. G. A. Pilkington, A. Oleshkevych, P. Pedraz, S. Watanabe, M. Radiom, A. B. Reddy, A. Vorobiev, S. Glavatskih, M. W. Rutland, Electroresponsive structuring and friction of a non-halogenated ionic liquid in a polar solvent: Effect of concentration. *Phys. Chem. Chem. Phys.* **22**, 19162–19171 (2020).

22. S. Glavatskih, E. Höglund, Tribotronics—Towards active tribology. *Tribol. Int.* **41**, 934–939 (2008).

23. G. A. Pilkington, R. Welbourn, A. Oleshkevych, S. Watanabe, P. Pedraz, M. Radiom, S. Glavatskih, M. W. Rutland, Effect of water on the electroresponsive structuring and friction in dilute and concentrated ionic liquid lubricant mixtures. *Phys. Chem. Chem. Phys.* **22**, 28191–28201 (2020).

24. G. A. Pilkington, K. Harris, E. Bergendal, A. B. Reddy, G. K. Palsson, A. Vorobiev, O. N. Antzutkin, S. Glavatskih, M. W. Rutland, Electro-responsivity of ionic liquid boundary layers in a polar solvent revealed by neutron reflectance. *J. Chem. Phys.* **148**, 193806 (2018).

25. S. Li, G. A. Pilkington, F. Mehler, O. S. Hammond, A. Boudier, A. Vorobiev, S. Glavatskih, M. W. Rutland, Tuneable interphase transitions in ionic liquid/carrier systems via voltage control. *J. Colloid Interface Sci.* **652**, 1240–1249 (2023).

26. C. C. A. Dreiss, Wormlike micelles: Where do we stand? Recent developments, linear rheology and scattering techniques. *Soft Matter* **3**, 956–970 (2007).

27. L. J. Magid, Z. Li, P. D. Butler, Flexibility of elongated sodium dodecyl sulfate micelles in aqueous salt solutions: A small-angle neutron scattering study. *Langmuir* **16**, 10028–10036 (2000).

28. N. C. Das, H. Cao, H. Kaiser, G. T. Warren, J. R. Gladden, P. E. Sokol, Shape and size of highly concentrated micelles in CTAB/NaSal solutions by small angle neutron scattering (SANS). *Langmuir* **28**, 11962–11968 (2012).

29. G. N. Smith, P. Brown, S. E. Rogers, J. Eastoe, Evidence for a critical micelle concentration of surfactants in hydrocarbon solvents. *Langmuir* **29**, 3252–3258 (2013).

30. A. Sanchez-Fernandez, A. E. Leung, E. G. Kelley, A. J. Jackson, Boundary layer thickness of non-halogenated ionic liquid lubricants in a polar solvent. *J. Colloid Interface Sci.* **579**, 794–804 (2020).

31. P. Jain, O. N. Antzutkin, 2-Ethylhexylsulfate anion-based surface-active ionic liquids (SAILs) as temperature persistent electrolytes for supercapacitors. *J. Ion. Liq.* **2**, 100034 (2022).

32. P. Jain, O. N. Antzutkin, Nonhalogenated surface-active ionic liquid as an electrolyte for supercapacitors. *ACS Appl. Energy Mater.* **4**, 7775–7785 (2021).

33. T. S. Bhat, P. S. Patil, R. B. Rakhi, Recent trends in electrolytes for supercapacitors. *J. Energy Storage* **50**, 104222 (2022).

34. K. C. S. Lakshmi, B. Vedhanarayanan, High-performance supercapacitors: A comprehensive review on paradigm shift of conventional energy storage devices. *Batteries* **9**, 202 (2023).

35. J. C. DeWitt, Ed., *Toxicological Effects of Perfluoroalkyl and Polyfluoroalkyl Substances* (Springer International Publishing, 2015).

36. R. Hayes, G. G. Warr, R. Atkin, Structure and nanostructure in ionic liquids. *Chem. Rev.* **115**, 6357–6426 (2015).

37. T. L. Greaves, C. J. Drummond, Solvent nanostructure, the solvophobic effect and amphiphile self-assembly in ionic liquids. *Chem. Soc. Rev.* **42**, 1096–1120 (2013).

38. B. Borah, G. R. Acharya, D. Grajeda, M. S. Emerson, M. A. Harris, A. M. M. Abeykoon, J. Sangoro, G. A. Baker, A. J. Nieuwkoop, C. J. Margulis, Do ionic liquids slow down in stages? *J. Am. Chem. Soc.* **145**, 25518–25522 (2023).

39. W. Clegg, A. J. Scott, F. J. Lawlor, N. C. Norman, T. B. Marder, C. Dai, P. Nguyen, Salts of the bis(catecholato)borate anion with organic cations. *Acta Crystallogr. C* **54**, 1875–1880 (1998).

40. N. Malviya, F. F. M. Yasin, M. T. Sateriale, F. Coleman, H. Q. N. Gunaratne, A. Dolfi, G. Srinivasan, M. Swadźba-Kwaśny, Task-specific boronium ionic liquids as ashless lubricant additives. *RSC Sustain.* **2**, 3100–3113 (2024).

41. H. Amir, M. Kar, L. A. O'Dell, M. Forsyth, M. Swadźba-Kwaśny, J. D. Holbrey, New tetrabutylphosphonium organic ionic plastic crystals incorporating borate anions. *J. Mater. Chem. A* **13**, 18842–18850 (2025).

42. C. P. Cabry, L. D'Andrea, K. Shimizu, I. Grillo, P. Li, S. Rogers, D. W. Bruce, J. N. C. Lopes, J. M. Slattery, Exploring the bulk-phase structure of ionic liquid mixtures using small-angle neutron scattering. *Faraday Discuss.* **206**, 265–289 (2018).

43. C. P. Cabry, L. D'Andrea, N. S. Elstone, S. Kirchhecker, A. Riccobono, I. Khazal, P. Li, S. E. Rogers, D. W. Bruce, J. M. Slattery, Small-angle neutron scattering from mixtures of long- and short-chain 3-alkyl-1-methyl imidazolium bistriflimides. *Phys. Chem. Chem. Phys.* **24**, 15811–15823 (2022).

44. A. Triolo, O. Russina, H. J. Bleif, E. Di Cola, Nanoscale segregation in room temperature ionic liquids. *J. Phys. Chem. B* **111**, 4641–4644 (2007).

45. Y. Zhang, J. B. Marlow, W. Millar, D. S. Silvester, G. G. Warr, H. Li, R. Atkin, Effect of ion structure on the nanostructure and electrochemistry of surface active ionic liquids. *J. Colloid Interface Sci.* **630**, 931–939 (2023).

46. B. Vestergaard, S. Hansen, Application of Bayesian analysis to indirect Fourier transformation in small-angle scattering. *J. Appl. Cryst.* **39**, 797–804 (2006).

47. S. Hansen, Bayesian estimation of hyperparameters for indirect Fourier transformation in small-angle scattering. *J. Appl. Cryst.* **33**, 1415–1421 (2000).

48. O. Glatter, G. Fritz, H. Lindner, J. Brunner-Popela, R. Mittelbach, R. Strey, S. U. Egelhaaf, Nonionic micelles near the critical point: Micellar growth and attractive interaction. *Langmuir* **16**, 8692–8701 (2000).

49. J. S. Pedersen, Analysis of small-angle scattering data from colloids and polymer solutions: Modeling and least-squares fitting. *Adv. Colloid Interface Sci.* **70**, 171–210 (1997).

50. J. B. Hayter, J. Penfold, An analytic structure factor for macroion solutions. *Mol. Phys.* **42**, 109–118 (1981).

51. J.-P. Hansen, J. B. Hayter, A rescaled MSA structure factor for dilute charged colloidal dispersions. *Mol. Phys.* **46**, 651–656 (1982).

52. M. C. Pedersen, S. L. Hansen, B. Markussen, L. Arleth, K. Mortensen, Quantification of the information in small-angle scattering data. *J. Appl. Cryst.* **47**, 2000–2010 (2014).

53. S. Li, *Electro-Interfacial Composition Control by Ionic Liquid Technology: Nanostructure, Self-Assembly, and Friction* (KTH Royal Institute of Technology, 2024).

54. R. G. Weiss, P. Terech, *Molecular Gels: Materials with Self-Assembled Fibrillar Networks* (Springer, 2006).

55. R. Abdel-Rahem, M. Gradzielski, H. Hoffmann, A novel viscoelastic system from a cationic surfactant and a hydrophobic counterion. *J. Colloid Interface Sci.* **288**, 570–582 (2005).

56. G. C. Kalur, S. R. Raghavan, Anionic wormlike micellar fluids that display cloud points: Rheology and phase behavior. *J. Phys. Chem. B* **109**, 8599–8604 (2005).

57. B. K. Mishra, S. D. Samant, P. Pradhan, S. B. Mishra, C. Manohar, A new strongly flow birefringent surfactant system. *Langmuir* **9**, 894–898 (1993).

58. K. Bijma, M. J. Blandamer, J. B. F. N. Engberts, Effect of counterions and headgroup hydrophobicity on properties of micelles formed by alkylpyridinium surfactants. 2. Microcalorimetry. *Langmuir* **14**, 79–83 (1998).

59. K. Bijma, J. B. F. N. Engberts, Effect of counterions on properties of micelles formed by alkylpyridinium surfactants. 1. Conductometry and1H-NMR chemical shifts. *Langmuir* **13**, 4843–4849 (1997).

60. P. Griffin, J. Kostal, Molecular simulations inform biomass dissolution in ionic liquids in pursuit of benign solvent-system design. *Green Chem.* **25**, 7283–7291 (2023).

61. M. Xu, W. Gao, C. Liu, P. Wei, H. Sun, Leveraging π-conjugation in benzimidazolium ionic liquids for selective extraction of bicyclic aromatic hydrocarbons: A combined experimental and computational study. *Energy Fuel* **39**, 19869–19883 (2025).

62. R. R. Hawker, J. B. Harper, in *Advances in Physical Organic Chemistry* (Elsevier, 2018), vol. 52, pp. 49–85.

63. W. Xu, L.-M. Wang, R. A. Nieman, C. A. Angell, Ionic liquids of chelated orthoborates as model ionic glassformers. *J. Phys. Chem. B* **107**, 11749–11756 (2003).

64. A. Sokolova, J. Christoforidis, A. Eltobaji, J. Barnes, F. Darmann, A. E. Whitten, L. de Campo, Bilby: Time-of-flight small angle scattering instrument. *Neutron News* **27**, 9–13 (2016).

65. A. Sokolova, A. E. Whitten, L. de Campo, J. Christoforidis, A. Eltobaji, J. Barnes, F. Darmann, A. Berry, Performance and characteristics of the Bilby time-of-flight small-angle neutron scattering instrument. *J. Appl. Cryst.* **52**, 1–12 (2019).

66. M. James, A. Nelson, S. A. Holt, T. Saerbeck, W. A. Hamilton, F. Klose, The multipurpose time-of-flight neutron reflectometer “Platypus” at Australia's OPAL reactor. *Nucl. Instrum. Methods Phys. Res. A* **632**, 112–123 (2011).

67. A. Devishvili, K. Zhernenkov, A. J. C. Dennison, B. P. Toperverg, M. Wolff, B. Hjörvarsson, H. Zabel, SuperADAM: Upgraded polarized neutron reflectometer at the Institut Laue-Langevin. *Rev. Sci. Instrum.* **84**, 025112 (2013).

68. M. Han, H. Kim, C. Leal, M. Negrito, J. D. Batteas, R. M. Espinosa-Marzal, Insight into the electrical double layer of ionic liquids revealed through its temporal evolution. *Adv. Mater. Interfaces* **7**, 2001313 (2020).

69. H. J. Hayler, S. Perkin, The eutectic point in choline chloride and ethylene glycol mixtures. *Chem. Commun.* **58**, 11204–11207 (2022).

70. O. Arnold, J. C. Bilheux, J. M. Borreguero, A. Buts, S. I. Campbell, L. Chapon, M. Doucet, N. Draper, R. F. Leal, M. A. Gigg, V. E. Lynch, A. Markvardsen, D. J. Mikkelson, R. L. Mikkelson, R. Miller, K. Palmen, P. Parker, G. Passos, T. G. Perring, P. F. Peterson, S. Ren, M. A. Reuter, A. T. Savici, J. W. Taylor, R. J. Taylor, R. Tolchenov, W. Zhou, J. Zikovsky, Mantid—Data analysis and visualization package for neutron scattering and μSR experiments. *Nucl. Instrum. Methods Phys. Res. A* **764**, 156–166 (2014).

71. J. Krzywon, P. Butler, SasView version 6.1.3 (Zenodo, 2026).

72. A. Glavic, M. Björck, GenX 3: The latest generation of an established tool. *J. Appl. Cryst.* **55**, 1063–1071 (2022).

73. A. Nelson, Co-refinement of multiple-contrast neutron/X-ray reflectivity data usingMOTOFIT. *J. Appl. Cryst.* **39**, 273–276 (2006).

74. D. Wakeham, A. Nelson, G. G. Warr, R. Atkin, Probing the protic ionic liquid surface using X-ray reflectivity. *Phys. Chem. Chem. Phys.* **13**, 20828–20835 (2011).
